# Suppression and Activation of Intracellular Immune Response in Initial Severe Acute Respiratory Syndrome Coronavirus 2 Infection

**DOI:** 10.3389/fmicb.2021.768740

**Published:** 2021-11-26

**Authors:** Lijia Jia, Zhen Chen, Yecheng Zhang, Li Ma, Liying Wang, Xiao Hu, Haizhou Liu, Jianjun Chen, Di Liu, Wuxiang Guan

**Affiliations:** ^1^Center for Bacteria and Viruses Resources and Bioinformation, Wuhan Institute of Virology, Center for Biosafety Mega-Science, Chinese Academy of Sciences, Wuhan, China; ^2^University of Chinese Academy of Sciences, Beijing, China; ^3^Center for Emerging Infectious Diseases, Wuhan Institute of Virology, Center for Biosafety Mega-Science, Chinese Academy of Sciences, Wuhan, China

**Keywords:** SARS-CoV-2, transcriptional dynamics, interferon response, host restriction factors, virus-host interactions

## Abstract

Severe acute respiratory syndrome coronavirus 2 (SARS-CoV-2) is currently the most important emerging pathogen worldwide, but its early transcriptional dynamics and host immune response remain unclear. Herein, the expression profiles of viral interactions with different types of hosts were comprehensively dissected to shed light on the early infection strategy of SARS-CoV-2 and the host immune response against infection. SARS-CoV-2 was found to exhibit a two-stage transcriptional strategy within the first 24 h of infection, comprising a lag phase that ends with the virus being paused and a log phase that starts when the viral load increases rapidly. Interestingly, the host innate immune response was found not to be activated (latent period) until the virus entered the log stage. Noteworthy, when intracellular immunity is suppressed, SARS-CoV-2 shows a correlation with dysregulation of metal ion homeostasis. Herein, the inhibitory activity of copper ions against SARS-CoV-2 was further validated in *in vitro* experiments. Coronavirus disease 2019-related genes (including CD38, PTX3, and TCN1) were also identified, which may serve as candidate host-restricted factors for interventional therapy. Collectively, these results confirm that the two-stage strategy of SARS-CoV-2 effectively aids its survival in early infection by regulating the host intracellular immunity, highlighting the key role of interferon in viral infection and potential therapeutic candidates for further investigations on antiviral strategies.

## Introduction

Coronavirus disease 2019 (COVID-19) is a novel pathology caused by the severe acute respiratory syndrome coronavirus 2 (SARS-CoV-2), which is highly infectious, insidious, and pathogenic. During viral infection, people often present with acute respiratory symptoms, such as fever, cough, fatigue, and dyspnea, and in severe cases, the disease develops into pneumonia, severe acute respiratory syndrome, renal failure, or even death (Guan et al., 2020; Zhou P. et al., 2020). COVID-19 was first discovered in Wuhan, China, at the end of 2019 and subsequently spread rapidly across the country and the world, causing a global pandemic. The World Health Organization reported 186,762,453 confirmed COVID-19 cases (including 4,030,918 deaths) as of July 13, 2021, which shows a grim situation of global prevention and control (WHO, 2020). In response to this outbreak, multiple types of vaccines were developed and administrated to people at an unprecedented rate (Dagotto et al., 2020; Wang et al., 2020), with 12.6% of the global population being fully vaccinated as of July 12, 2021.^1^ The successful development of these vaccines is a leap forward in COVID-19 research, but given the time required to establish the immune barrier and the continued emergence of new virus variants, SARS-CoV-2 will continue to impact human society for some time in the future. Therefore, it is necessary to continue to explore the etiology and infection characteristics of SARS-CoV-2.

Severe acute respiratory syndrome coronavirus 2 is a beta genus coronavirus that has a genome of nearly 30-kb single-stranded RNA with a 5′ cap structure and 3′ poly-A tail, encoding structural [spike (S), envelope (E), membrane (M), and nucleocapsid (N) proteins] and accessory (3a, 6, 7a, 7b, 8, and 10) proteins, as well as non-structural proteins (nsp 1–16) (Gorbalenya et al., 2020; Kim et al., 2020; Wu et al., 2020). Several studies have revealed the structure of the viral genome and transcriptome (Kim et al., 2020), the three-dimensional structure of the viral particles, and the functions of important viral proteins (Bojkova et al., 2020; Yao H. et al., 2020). However, the expression patterns and dynamics of SARS-CoV-2 viral genes remain unclear, and whether they differ within cells with different physiological functions has not been elucidated.

Although no specific therapy for SARS-CoV-2 has been identified to date, some generic antivirals may be effective in COVID-19 treatment, such as type I interferon (IFN), a cytokine that plays an important antiviral role in innate immunity (Hung et al., 2020; Sa Ribero et al., 2020). In contrast to the inadequate IFN responses in SARS patients (Reghunathan et al., 2005; Ziegler et al., 2005), a robust IFN-directed antiviral response was observed in patients with COVID-19, even leading to cytokine storm that exacerbates the disease when pro-inflammatory cytokines were produced in excess (Yao Z. et al., 2020; Zhou Z. et al., 2020). Hence, lack of type I IFN may be a hallmark of severe COVID-19 (Hadjadj et al., 2020). Indeed, a recent study further reported that at least 10.2% of 987 patients with life-threatening COVID-19 pneumonia had human inborn errors in IFNs (existing autoantibodies against type I IFNs) (Bastard et al., 2020), demonstrating the strong link between IFN and SARS-CoV-2 infection.

In the present study, we compared Vero E6 cells, a commonly used monkey kidney cell line for preparation and propagation of SARS-CoV-2 (Harcourt et al., 2020), with Huh7 cells, a human hepatocyte cell line insensitive to SARS-CoV-2, to characterize early transcriptional dynamics of the virus and the respective response landscape in the two cell lines. In addition, the response to SARS-CoV-2 of Huh7 cells and its derivative cell line Huh7.5.1 (type I IFN deficient) was investigated to explore the potential role of IFN in the early stages of SARS-CoV-2 infection.

## Materials and Methods

### Facility and Ethics Statements

All experiments related to living SARS-CoV-2 virus were performed under biosafety level 3 (BSL-3) conditions at a negative pressure in Wuhan Institute of Virology, Chinese Academy of Sciences.

### Cell Lines and Culture

Huh7 and Huh7.5.1 hepatocytes were obtained from the Wuhan Institute of Virology of the Chinese Academy of Sciences. Vero E6 cells (American Type Culture Collection # CRL1586) were purchased from the American Type Culture Collection. All cells were cultured in Dulbecco’s modified Eagle’s medium (DMEM) containing 10% fetal bovine serum and incubated at 37°C under a condition of 5% CO_2_.

### Virus Strains and Infection

Severe acute respiratory syndrome coronavirus 2 (Genbank: NC_045512.2) was propagated on the Vero E6 cells and titrated by a single layer plaque assay with standard procedure. Briefly, Vero E6 cells were seeded into 24-well plates at a concentration of 1 × 10^5^ cells per well. Twenty-four hours later, confluent Vero E6 cells were infected with 200 μl of DMEM containing a serial 10-fold dilution of viral stock for 1 h at 37°C. After removal of the inoculum, Vero E6 cells were overlaid with DMEM containing 0.9% methylcellulose and cultured at 37°C for 4 days. Plaques were monitored and counted.

Because cells differ in their susceptibility to SARS-CoV-2, to make viral transcription in cells observable, we tested different gradients of viral multiplicity of infection (MOI) and finally determined the minimum MOI for effective infection in each cell. Then, SARS-CoV-2 virus infections were performed in Huh7, Vero E6, and Huh7.5.1 cells using their minimum effective MOI of 0.5, 0.05, and 0.05, respectively. Cells were collected from 0 to 24 hpi and lysed with 1-ml TRIzol reagent (Invitrogen) to obtain total final RNA for RNA sequencing after every 2 h.

### Immunofluorescence

To visualize SARS-CoV-2-infected cells, the infected cells at the indicated time were fixed twice in 4% formaldehyde in PBS and washed twice in PBS. After permeabilization with 0.1% Triton X-100, the cells were stained with a monoclonal antibody recognizing SARS-CoV-2 N protein. The bound antibodies were visualized by using Alexa Fluor 488-labeled (green) goat anti-rabbit immunoglobulin G.

### Next-Generation Sequencing Library Preparation and Sequencing

RNAs from infected cells were quantified separately using Qubit 3.0, and qualified samples were further enriched for messenger RNA (mRNA) using Dynabeads mRNA Purification Kit (Invitrogen, cat. no. 61006), followed by fragmentation, reverse transcription, and double-stranded complementary DNA synthesis. We ligated the adaptor at the end of the repaired double-stranded complementary DNA and cyclized the polymerase chain reaction (PCR) products using MGIEasy mRNA Library Prep Kit (no. 85-05536-01, MGI Technology, Shenzhen, China) according to the manufacturer’s instructions to obtain the final libraries. After detection by the Agilent 2100 Bioanalyzer, the libraries were loaded into the chip of the MGI2000 platform for paired-ends sequencing based on DNA nano ball technology, with a read length of 150 bp.

### Raw Data Processing

The raw data were first quality filtered using fastp (v0.20.1) (Chen et al., 2018) to trim 10 low-quality bases at the front and tail of the sequences, removing sequences with shorter than 20 bases and reads with a number of N bases > 6. Clean reads were compared with the host and viral reference genomes by hisat2 (v2.1.0) (Kim et al., 2015) (Vero E6: http://ftp.ensembl.org/pub/release-100/fasta/chlorocebus_sabaeus/dna/Chlorocebus_sabaeus.ChlSab1.1.dna.toplevel.fa.gz; Huh7 and Huh7.5.1: http://ftp.ensembl. org/pub/release-100/fasta/homo_sapiens/dna/Homo_sapiens.GRCh38.dna.primary_assembly.fa.gz; SARS-CoV-2: NC_045512.2); samtools (v1.9) (Li et al., 2009) was used for process file format conversion and mapping rate statistics. Based on the genome annotation (Vero E6: http://ftp.ensembl.org/pub/release-100/gtf/chlorocebus_sabaeus/Chlorocebus_sabaeus.ChlSab1.1.100.chr.gtf.gz; Huh7 and Huh7.5.1: http://ftp.ensembl.org/pub/release-100/gtf/homo_sapiens/Homo_sapiens.GRCh38.100.chr.gtf.gz; SARS-CoV-2: homemade) and the resulting BAM files, the raw gene count matrices for each time point of Vero, huh7, and SARS-CoV-2 contained in them were obtained independently by featureCounts (v1.6.3) (Liao et al., 2014) for subsequent analysis.

### Differential Expression Analysis

DESeq2 (v1.26.0) (Love et al., 2014) was used in this study to analyze differential gene expression. Gene count matrices for Vero E6, Huh7, and Huh7.5.1 were used as input files to explore the differential expression of host genes as SARS-CoV-2 infection became longer, using their respective 0 hpi as a control group with a *p*-value < 0.05 and | log2fc| > 1 as the criterion. Based on the K-medoid algorithm, the identified differentially expressed genes (DEGs) were clustered into clusters with different expression patterns. The graphical part was implemented by ggplot2 (v3.3.2) (Wickham, 2009). In addition, some important DEGs such as cytokines and DEGs shared by two cell lines were highlighted in the form of heat maps.

### Functional Enrichment Analysis

Gene ontology (GO) functional analysis and Kyoto Encyclopedia of Genes and Genomes (KEGG) pathway analysis were applied to the multiple DEG sets. Multiple hypothesis testing corrections for a *p*-value was performed by clusterProfiler (v3.14.3) (Yu et al., 2012) using the Benjamini and Hochberg false discovery rate method, retaining only GO entries and KEGG pathways with an adjusted *p* < 0.05. The concept of z-score, as mentioned by GOplot (v1.0.2) (Walter et al., 2015), was used here to characterize whether biological processes are more likely to be decreased (negative value) or increased (positive value), with the formula $z-s\,c\,o\,r\,e=\frac{\left(u\,p-d\,o\,w\,n\right)}{\sqrt{c\,o\,u\,n\,t}}$. The top 10 GO terms independently enriched at each time point were presented by Circos (v0.69.6) (Krzywinski, 2009), whereas the semantic similarity between terms was computed by GOSemSim (v2.12.1) (Yu et al., 2010).

### Gene Expression of Severe Acute Respiratory Syndrome Coronavirus 2

Appropriate host internal reference genes are essential for standardizing the expression levels of viral genes, so we first calculated the coefficient of variation (${\text{c}}_{v},{\text{c}}_{v}=\frac{\sigma }{\mu }$) of the host genes to assess their stability and selected the three most stable of them as reference genes (ref-genes, RGs). The viral expressions were normalized by the formula “$G_{\mathrm{normalized}\,\mathrm{counts}}=\frac{G_{\mathrm{raw}\,\mathrm{counts}}}{G_{\text{length}}*\sqrt[{3}]{\prod_{i=1}^{3}{\text{refgene}}_{\text{i}}}}$” as input to plot the expression curve. The growth rate (*G*_*growth  rate*_, $G_{\mathrm{growth}\,\mathrm{rate}}=\frac{\Delta \,G_{\mathrm{normalized}\,\mathrm{counts}}}{\Delta \,\text{t}}$) of viral genes during infection was further calculated to investigate the pattern of changes in the transcriptional activity of each gene.

### Construction of Weighted Co-expression Networks

The weighted gene co-expression networks were constructed by R package *WGCNA* (v1.69) (Zhang and Horvath, 2005). The full gene expression matrices of Vero E6 and Huh7 after variance stabilizing transformation standardization were read, respectively, and the one-step network construction was performed with β value = 6 and cut height = 0.25 to make the gene distribution conform to a scale-free network. The time of infection of the cell samples was used as trait information, and the most relevant gene modules were identified by Pearson correlation analysis. The genes contained in each module were subjected to GO enrichment analysis by clusterProfiler, and the modules most relevant to SARS-CoV-2 infection were further extracted into Cytoscape (v3.7.2) (Shannon et al., 2003) for visualization and extracted hub-genes using the cytoHubba plugin.

### Discriminatory Role of Hub-Genes in Clinical Data

In the face of SARS-CoV-2 infection, DEGs shared by two different cell lines and hub-genes of infection-associated modules in Huh7 were considered as candidate markers to identify COVID-19. We used a random forest algorithm (R package *randomForest* v4.16.14) to screen for biomarkers that best distinguish clinical COVID-19 patients from non-COVID-19 patients and evaluated the classification effect by receiver operating characteristic (ROC) curves and area under the ROC curve. Large-scale clinical patient data were obtained from the Gene Expression Omnibus public database, including 100 COVID-19 and 26 non-COVID-19 inpatients at Albany Medical Center from April to June 2020 (Gene Expression Omnibus accession: GSE157103) (Overmyer et al., 2021). According to the literature, the team used LeukoLOCK^®^ filters to process patient blood samples to obtain leukocytes and further eluted RNA for RNA-seq (Overmyer et al., 2021).

### Addition of Copper Ion and Cytotoxicity Assay

Vero E6 cells were seeded in 96-well plates (1 × 10^4^ cells per well) and allowed to grow for 24 h before treatment. Then, serial 10-fold dilutions of copper sulfate were added to the cells. At 48 h, the cells were incubated with a 10-μl CCK-8 reagent (cell counting kit-8, Bimake) for 1 h at 37°C. The absorbance at 450 nm was read by a microplate reader (Varioskan Flash, Thermo Fisher). For each compound concentration, six wells were performed in parallel, and the mean values of the cell viability were calculated. The inhibition activity of copper ions on cell viability was expressed as a percentage of the treated cells to untreated cells. Using non-linear regression analysis of GraphPad Prism 8.0 software, the cytotoxic concentration of CC_50_, at which 50% of the cells are viable, was calculated.

After determining the cytotoxicity of copper ion, Vero E6 cells and Huh7 cells were cultured in the standard medium or the medium supplemented with different concentrations of CuSO_4_ (a concentration of 0.125, 0.25, or 0.5 mM). Then, SARS-CoV-2 (MOI = 0.05 for Vero-E6 cells and MOI = 0.5 for Huh7 cells) was added to each well, incubated at 37°C for 24 h, and the viral genes N and RdRp in the supernatant were measured by relative real-time quantitative (Qrt)-PCR assay (N-QF: 5′- TAACCAGAATGGAGAACGCAGTG-3′, N-QR: 5′-TGAGTGAGAGCGGTGAACCAAGAC-3′; RdRp-QF: 5′-CAAAATGYTGGACTGAGACTGACC-3′, RdRp-QR: 5′-ACGATATCATCDACAAAACAGCCG-3′).

## Results

### Distinct Transcriptional Dynamics of Severe Acute Respiratory Syndrome Coronavirus 2 in Sensitive and Unsensitive Cell Lines

**FIGURE 1:**
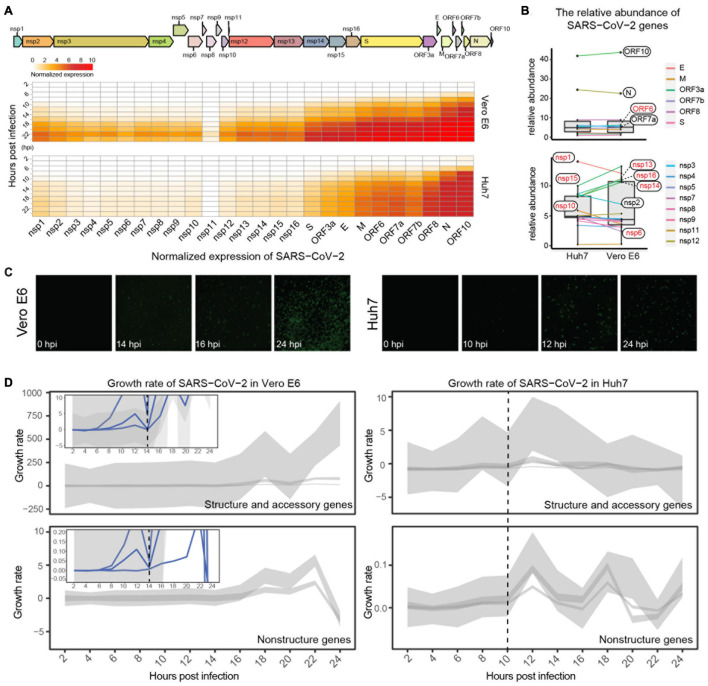
Expression dynamics of SARS-CoV-2 viral genes. **(A)** Genomic structure of SARS-CoV-2 and expression trends of its genes in Vero E6 and Huh7. Heatmap showing dynamics of normalized viral gene expression (ranging from 0 to 10), with white and red representing no and high expression, respectively, and horizontal and vertical axes representing each gene of SARS-CoV-2 and time of infection, respectively. **(B)** Comparison of relative abundance of each viral gene in different cells. Mean value of each viral gene at 0–24 hpi was calculated, and its percentage of total expression was determined as relative abundance; genes with inconsistent trends are marked in red. **(C)** Immunofluorescence assay of nucleocapsid protein of SARS-CoV-2. **(D)** Growth pattern of viral genes in Vero E6 and Huh7 cells. Expression heatmaps consist of normalized counts, which show accumulation of viral load, and growth curves represent transcriptional activity of viral genes at different moments. Due to large variation in y-axis range, structural genes are shown separately from non-structural genes, and small views of same y-axis range as Huh7 are appended to Vero E6.

To elucidate the transcriptional dynamics of SARS-CoV-2 in Vero E6 and Huh7 cell lines, we first standardized the viral gene counts by using host reference genes (*BMS1*, *IWS1*, and *WDR33* for Huh7 and *PPP2CB*, *SEL1L3*, and *HADH* for Vero E6) and investigated the accumulation pattern of viral genes after infection. Overall, the expression of viral genes was much higher in Vero E6 than in Huh7 from 8 hpi, although both showed an increasing trend (Figure 1A, Supplementary Figures 1A,B, and Supplementary Table 1). Using the SARS-CoV-2-sensitive Vero E6 as control, the relative abundance of each viral gene was roughly stable in Huh7, except for decreased *ORF6*, *nsp13*, *nsp14*, *nsp15*, and *nsp16*, and increased *nsp1*, *nsp6*, and *nsp10*. The genes with the highest and lowest abundance were both *ORF10* and *S*, respectively (Figure 1B). As Vero E6 has a defective type I IFN secretion and ORF6, nsp13, nsp14, and nsp15 have recently been shown to be potent IFN antagonists (Yuen et al., 2020), it is possible that they exhibit lower accumulation in Huh7 because of their interactions with IFN, whereas the interactions of *nsp1*, *nsp6*, and *nsp10* with IFN are still unknown.

Immunofluorescence imaging targeting viral N proteins was used next to confirm the viral transcriptional dynamics. A gradual increase in viral proteins in Vero E6 from 14 hpi was observed, with more than 70% of cells being invaded at 24 hpi (Figure 1C and Supplementary Figure 1E); however, the increase in N proteins in Huh7 started at 12 hpi, and only less than 10% of cells were invaded at 24 hpi. These results indicate that the transcription efficiency of SARS-CoV-2 differed between the two cell lines. Further calculation of the transcriptional activity of the viral genes revealed that the transcriptional activity of all viral genes in Vero E6 reached a small peak at 12 hpi and decreased to zero at 14 hpi, when the genes had completed the first round of active transcription and then entered the second round of active state (Figure 1D and Supplementary Figures 1C,D). Unexpectedly, the first active cycle of viral genes ended at 10 hpi in Huh7, which was earlier than in Vero E6 (Figure 1D).

### Differential Responses Against Severe Acute Respiratory Syndrome Coronavirus 2 in Vero E6 and Huh7

**FIGURE 2:**
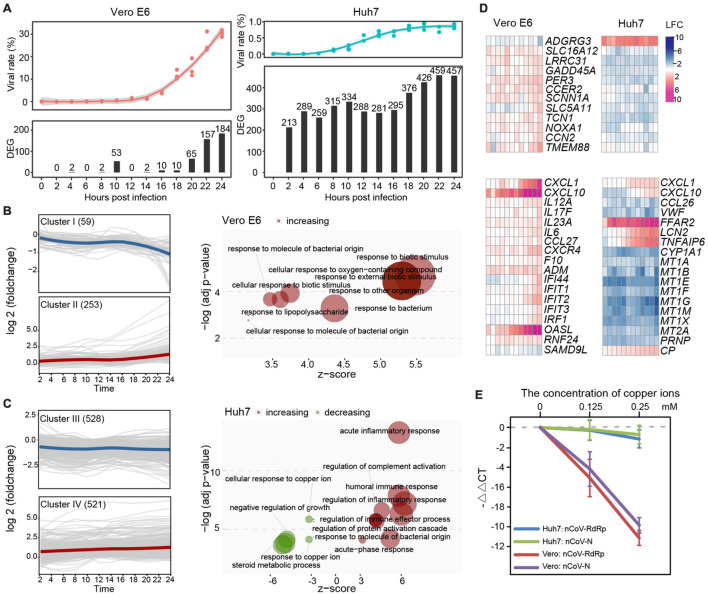
Comparative analysis between SARS-CoV-2-induced response on Vero E6 and Huh7 cells during 24 h. **(A)** Proportion of SARS-CoV-2 and distribution of DEGs in two cell lines during infection. **(B,C)** Functionality of DEG sets with different expression patterns in Vero E6 and Huh7, respectively. Unsupervised clustering based on k-medoids algorithm was performed on DEGs of two cell lines, and resulting clusters were subjected to GO enrichment analysis. Downregulated gene set cluster I in Vero E6 was not enriched for functional terms with *P* < 0.05. **(D)** Noteworthy characteristics of DEGs are highlighted. **(E)** SARS-CoV-2 viruses were suppressed by addition of different concentrations of copper ions *in vitro*. *RdRp* and *N* of SARS-CoV-2 in cell culture supernatant were detected separately, and –ΔΔCt (i.e., log2foldchange) was calculated using blank group as control.

Possibly due to the deficient pathway of type I IFN, only a few genes were differentially expressed in Vero E6 during the first 20 h of infection and increased at 22–24 hpi, when the mapping rate of SARS-CoV-2 was already as high as 26.10–30.27% (Figure 2A and Supplementary Table 1). In contrast, a considerable number of DEGs at each time point, with only a small decrease at 12–16 hpi were observed in Huh7. qRT-PCR was also performed on a small number of genes to confirm the reliability of the RNA-Seq results (Supplementary Figure 2A and Supplementary Table 2). Based on the expression pattern, 312 and 1,049 DEGs of Vero E6 and Huh7, respectively, were clustered into downregulated clusters I and III and upregulated clusters II and IV (Figures 2B,C). Functional enrichment of the four clusters showed that biological processes related to defense against exogenous bacteria were increased in both cell lines. In addition, processes associated with acute inflammatory responses were increased in Huh7 cells, whereas processes associated with metal ions were inhibited (Figures 2B,C and Supplementary Table 2).

Next, detailed analysis of the 49 DEGs shared by Vero E6 and Huh7 cells, which may be generic genes associated with SARS-CoV-2 infection, revealed that some of these genes, which were related to positive regulation of cell death, had opposite expression trends in the two cell lines (Figure 2D and Supplementary Figure 2B). Moreover, SARS-CoV-2 infection of renal and hepatocyte cells was found to potentially trigger a wave of inflammatory responses accompanied by increased expression of pro-inflammatory cytokines and immune markers (Supplementary Figure 2C). Although neither Vero E6 nor Huh7 cells are immune cells and several common checkpoint genes were not significantly altered, many cytokine-related genes, including chemokines, interleukins (ILs), tumor necrosis factor (TNF), and IFN, were upregulated upon viral stimulation, suggesting the presence of a cytokine storm. We found that these signaling molecules could be divided into two categories, according to their expression patterns: genes that were continuously upregulated during the time of infection, such as *CXCL10*, *ACKR4*, *CX3CR1*, *OASL*, and *ADM* in Vero E6 and *CXCR6*, *CXCL2*, *CCL27*, *TNFSF11*, *TNFSF18*, *FFAR2*, *STEAP4*, *APOBEC3G*, *CEBPD*, *LRG1*, *CRP*, and *SAA1* in Huh7 (Supplementary Figure 2C), and genes that respond during the second stage of the infection, such as *CXCL1*, *CXCL6*, *IL6*, *TNFAIP6*, *C1S*, and *LCN2*. *CXCL10*, *CXCL1*, and *OASL* were the most upregulated genes in Vero E6 (Figure 2D). Indeed, *CXCL10* was reported to be a key regulator of the cytokine storm in SARS-CoV-2 infection, *CXCL1* to play a role in chemotactic neutrophils in bacterial infections, and *OASL* to be involved in IFN-gamma signaling and PI3K-Akt signaling pathways, which can restrict virus infection (Stelzer et al., 2016). In Huh7 cells, increased expression of acute-phase proteins, such as C-reactive protein and serum amyloid A1, was observed with the progression of infection, whereas *FFAR2* and *STEAP4* with metalloreductase activity showed significantly higher upregulation than other genes (Figure 2D and Supplementary Table 2B). Similar to Vero, a number of cytokines associated with bacteria were identified in Huh7 cells, such as lipocalin 2 (*LCN2*), which inhibits bacterial infection, was upregulated, whereas C-C motif chemokine ligand 26 (*CCL26*), a chemoattractant for eosinophils and basophils, was downregulated.

In addition, many of the downregulated DEGs in Huh7 belonged to the metallothionein gene family, which led us to investigate further the role of metal ions, especially copper ions, in SARS-CoV-2 infection. *In vitro* experiments confirmed that the addition of copper ions into the cell culture could effectively inhibit SARS-CoV-2 replication in a dose-dependent manner, but the inhibition rate of copper ions against the virus in Vero E6 cells was much higher than that in Huh7 (Figure 2E).

### Severe Acute Respiratory Syndrome Coronavirus 2 Does Not Elicit Significant Cellular Immune Response Until the Second Cycle of Transcription in the Host

**FIGURE 3:**
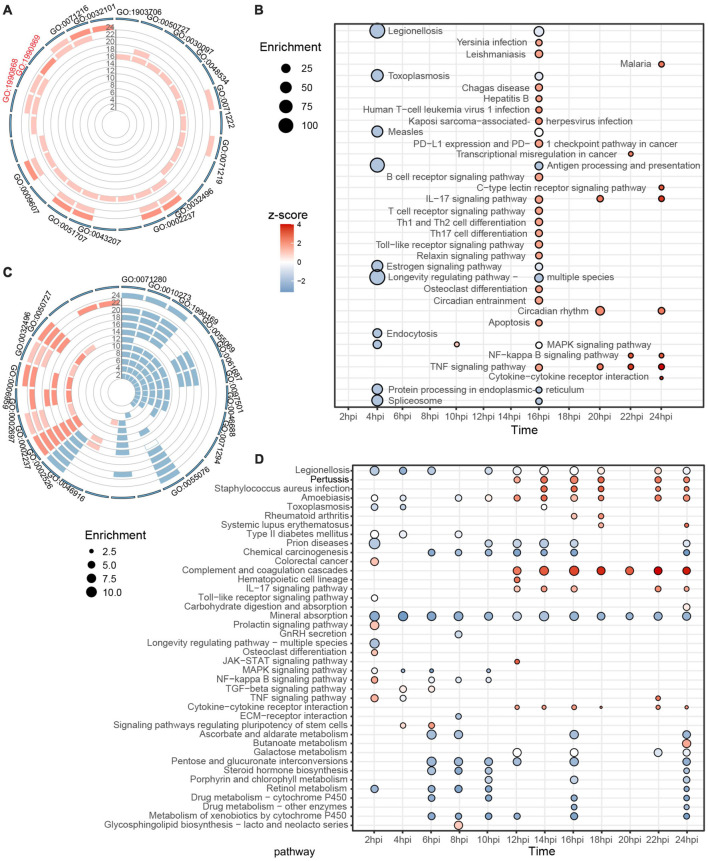
Individual GO and KEGG enrichment analysis based on identified DEGs. **(A,B)** Dynamic biological processes and pathways in Vero E6 between 2 and 24 hpi, with top 10 terms displayed for each time. **(C,D)** Similarly, trends in biological processes and pathways in Huh7 during SARS-CoV-2 infection. Color of bar chart or bubble represents *z*-score (up-/down trend); size of bubble represents enrichment factor. All results meet *P* < 0.05, sorted by *P*, and top 10 results from each group are shown. See Supplementary Table 3 for additional details.

Gene ontology and Kyoto Encyclopedia of Genes and Genomes analyses by individual time-point were conducted to probe the host immune response during the two-stage SARS-CoV-2 infection process. In the case of Vero E6, biological processes associated with hematopoiesis, inflammation, and immune response were gradually enriched and upregulated from 16 hpi but without any significant changes during the first 14 h (Figure 3A and Supplementary Table 3A). Defensive processes against bacterial infections, such as cellular response to lipopolysaccharide (GO:0071222), response to molecule of bacterial origin (GO:0002237), and response to a bacterium (GO:0009617), were repeatedly enriched. Accordingly, KEGG analysis also showed no significant differences in cellular pathways. Only small-scale pathways were downregulated in the first 14 hpi, and multiple infectious disease and immune-related pathways were activated after 16 hpi (Figure 3B).

Unlike Vero E6, many biological processes were altered in Huh7 cells after SARS-CoV-2 infection. Combined with GO-terms semantic similarity analysis, the predominant state of Huh7 between 2 and 10 hpi was found to be the inhibition of metal ion-related processes, such as cellular response to copper ion (GO:0071280), detoxification of copper ion (GO:0010273), zinc ion homeostasis (GO:0055069), and stress response to metal ion (GO:0097501), whereas within 12–24 hpi, cellular immune-related responses were activated, such as acute inflammatory response (GO:0002526), response to molecule of bacterial origin (GO:0002237), humoral immune response (GO:0006959), and regulation of inflammatory response (GO:0050727) (Figure 3C and Supplementary Figure 3). KEGG analysis provided consistent results, with most of the enriched pathways associated with metal ions being downregulated in the first 10 h, and active immune-related responses being noticeable after 12 hpi, including infectious diseases (associated with bacteria and parasites), complement and coagulation cascades, IL-17 signaling pathway, and cytokine–cytokine receptor interaction (Figure 3D). Overall, neither Vero E6 nor Huh7 showed a corresponding rapid antiviral response during the first round of active viral transcription. After the virus entered the second stage of transcription, the results showed an enrichment of immune pathways associated with bacterial or even parasitic infections and inflammatory responses mediated by IL-17.

### Network of Co-expressed Gene Modules Associated With Viral Infection

**FIGURE 4:**
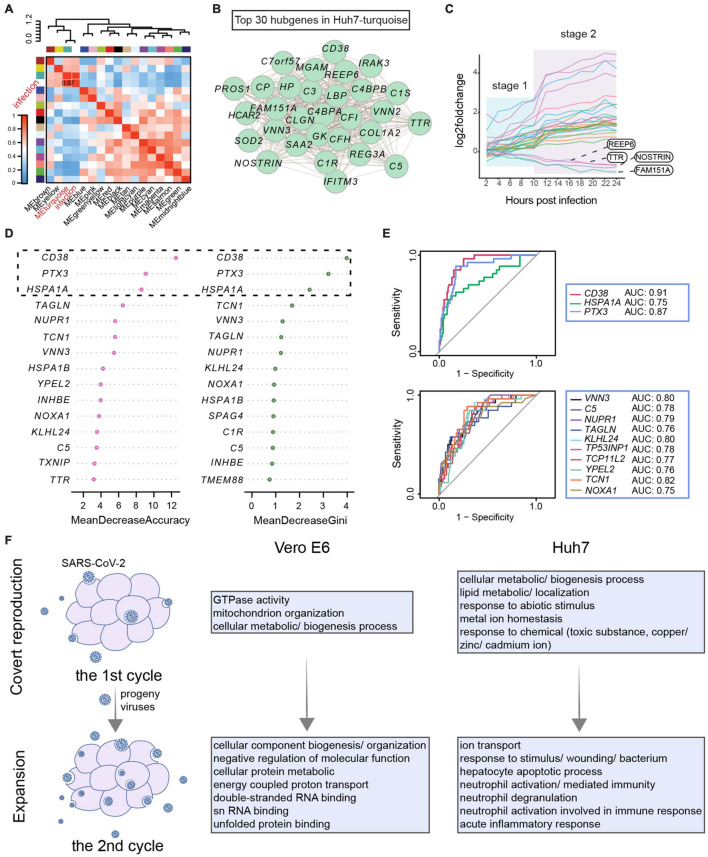
Developmental patterns of co-expression gene modules associated with SARS-CoV-2 infection. **(A–C)** Weighted gene co-expression network analysis for Huh7. **(A)** Pearson correlation of gene modules with SARS-CoV-2 infection. **(B)** Top 30 hub genes in turquoise module associated with infection. **(C)** Stage-related changes in expression trends of 30 hub genes occurring after 10 hpi. **(D,E)** Classification model to identify COVID-19 based on core genes. **(D)** Characterization of core genes in clinical patients. Expression patterns of 79 key genes in COVID-19 and non-COVID-19 patients were evaluated. Then, patients were classified by a random forest algorithm, and classification contribution of each gene was assessed (MeanDecreaseAccuracy and MeanDecreaseGini). Top 15 genes achieved most effective identification are shown here. **(E)** Receiver operating characteristic curves for genes with good identification of COVID-19 patients. True-positive rate (sensitivity) is represented as vertical coordinate and false-positive rate (1-specificity) as horizontal coordinate. In combination with area under receiver operating characteristic curve, classification effects of core genes were evaluated. **(F)** Host response pattern differs between first and second rounds of viral infection. Functional enrichment analysis of co-expression modules of genes most affected by virus in first and second rounds of infection reveals cellular state.

Considering the subjective effect of selecting DEGs by a threshold, we used all host genes to construct weighted gene co-expression networks. The 8,415 genes of Vero E6 were clustered into 11 modules, and the yellow module was associated with double-stranded DNA binding and transcriptional regulation, showing the highest correlation with the SARS-CoV-2 infection process (Supplementary Figures 4A–C and Supplementary Table 4). The core genes of the yellow module encoded enzymes or binding proteins required for transcriptional regulation and basic substance generation, including *FABP5* related to fatty acid uptake, transport, and metabolism and *THBS1* involved in endoplasmic reticulum stress (Supplementary Figures 4D,E). The 12,894 genes of Huh7 were grouped into 17 clusters, of which the turquoise module was strongly associated with viral infection events (Figure 4A and Supplementary Figures 4F–H). Genes in the turquoise module were indeed associated with acute inflammatory response and were involved in the complement system (*C4BPA*, *C3*, *C1R*, *C1S*, *CFH*, *C5*, and *C4BPB*), coagulation (*PROS*), and innate immunity (*SAA1*, *HP*, *IFITM3*, and *LBP*) (Figures 4B,C).

To clarify whether these infection-related immune genes were specifically affected by SARS-CoV-2, we examined the expression of shared DEGs and infection-associated hub genes in leukocytes from clinical patients (Figure 4B, Supplementary Figure 2B, and Supplementary Table 5). These clinical patients consisted of non-COVID-19 donors (*n* = 26) and COVID-19 patients (*n* = 100) presenting from mild to critical disease grades (Supplementary Figure 5A; Overmyer et al., 2021). These genes were used as identifiers to classify the 126 patients based on the random forest algorithm, revealing that *CD38*, *PTX3*, and *HSPA1A* were able to classify patients into the correct groups and contribute specifically to COVID-19 (Figure 4D). Analysis of the ROC further showed that *CD38*, *PTX3*, and *TCN1* performed well with an area under the ROC curve greater than 0.8. In particular, *CD38* was found to distinguish COVID-19 patients at the RNA level; thus, it may serve as a potential host target for antiviral research (Figure 4E and Supplementary Figure 5B).

Overall, the trend of the hub genes showed a two-stage profile corresponding to the transcriptional stage of SARS-CoV-2 within 24 h (Figure 4C and Supplementary Figure 4E). Therefore, weighted gene co-expression network analysis was performed next to assess in detail each stage of viral infection. In the first transcriptional stage of the virus, the enriched biological processes were mainly cellular metabolic and biogenesis processes, as well as lipid and metal ion homeostasis in Huh7 (Figure 4F and Supplementary Figures 6A–D, 7A–D). In the second stage, the most affected pathways in Vero E6 were the metabolism and binding of macromolecules, such as proteins and double-stranded RNA, which may be related to the binding of the host inflammatory sensor to intermediates of viral nucleic acids (Bauernfried et al., 2020). When the virus finished its first stage and began the second stage, immune processes related to response to stimulus, neutrophil-mediated immunity, and acute inflammatory response became apparent in Huh7 cells (Figure 4F and Supplementary Figures 6E–H, 7E–H). Hence, the host’s innate immune system is activated during the second stage. These results indicated that the difference in immune response at different time points might be due to the two-stage strategy of SARS-CoV-2.

### Effect of Type I Interferon on the Interaction Between Hepatocytes and Severe Acute Respiratory Syndrome Coronavirus 2

**FIGURE 5:**
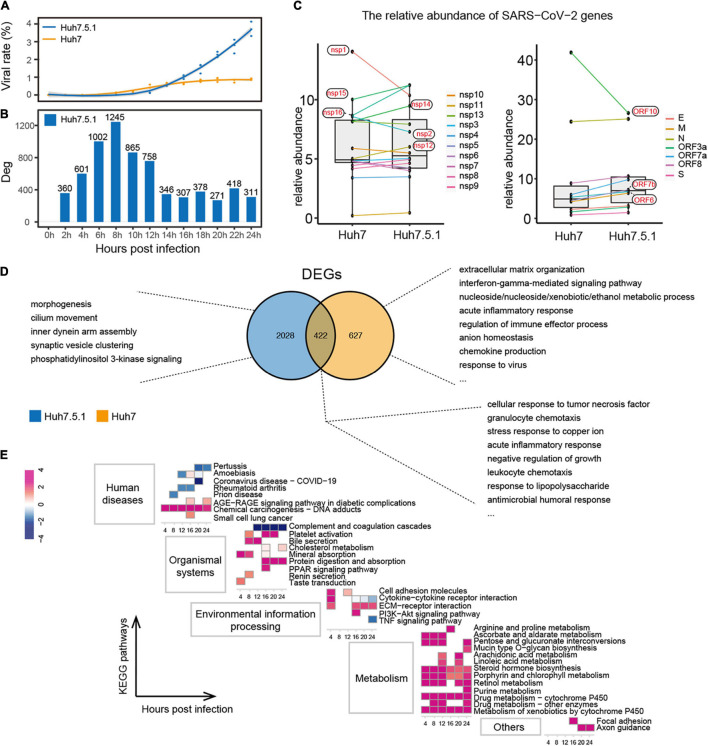
Interaction of IFN+/– hepatocytes with SARS-CoV-2. **(A,B)** Proportion of SARS-CoV-2 **(A)** and distribution of DEG numbers **(B)** in Huh7.5.1 during infection. **(C)** Comparison of relative abundance of each viral gene in IFN+/– hepatocytes. **(D)** Functions of unique and shared DEGs within Huh7.5.1 and Huh7 cells. **(E)** Enriched pathways in Huh7.5.1 are shown along with corresponding Huh7 at each time point as a control. Colors represent *z*-score of the pathways.

Next, to explore the role of IFN in the two stages of SARS-CoV-2 infection, we established infection experiments with Huh7.5.1, a type I IFN-deficient cell line of Huh7, and compared them based on the responses of the virus and the host. The proportion of virus in Huh7.5.1 was found to be as low as that in Huh7 in the first stage of viral infection, but its increase rate from 14 hpi onward was much higher than that in Huh7, reaching approximately 3.72% by 24 hpi (Figure 5A). In addition, the transcriptional dynamics of the viral genes showed that the first transcriptional stage of SARS-CoV-2 in Huh7.5.1 was 0–12 h (Supplementary Figures 8A,B), indicating that SARS-CoV-2 efficiently replicated from the second round of infection. Interestingly, the transcriptional stage of the virus was positively correlated with the susceptibility of the cell to the virus, with the more susceptible cells (Vero E6 > Huh7.5.1 > Huh7) showing increased viral replication and a longer transcriptional stage. We further compared the abundance of each viral gene in both cell lines. Compared with Huh7.5.1, the abundance of *ORF6*, *nsp14*, *nsp15*, and *nsp16* was decreased, and the expression of *nsp1* was increased in Huh7 cells, which is consistent with previous results (Figure 1B). In addition, *nsp2* and *ORF10* were increased in Huh7, whereas *nsp12* and *ORF7b* were decreased in Huh7 cells (Figure 5C).

Unlike Huh7, Huh7.5.1 produced more DEGs in response to SARS-CoV-2 in the first stage of viral infection; however, these DEGs still did not trigger an antiviral immune response (Figure 5B and Supplementary Figures 8C,D). The intracellular immune activity of IFN-normal Huh7 was significantly affected; unique DEGs of which were mainly involved in antiviral responses, such as IFN-gamma-mediated signaling pathway, acute inflammatory response, chemokine production, and response to the virus. In contrast, the functions of DEGs unique to IFN-deficient Huh7.5.1 were related to basal morphogenesis (Figure 5D). Nonetheless, the DEGs shared by Huh7 and Huh7.5.1 cells represented a generalized pattern of hepatocyte-SARS-CoV-2 interactions, primarily including an antimicrobial immune response and stress response to copper ions. Finally, Huh7 cells were used as the control group to integrate the datasets of Huh7 and Huh7.5.1, further showing that immune pathways in Huh7.5.1 cells were downregulated, whereas pathways associated with cancer response, metal ion homeostasis, and especially metabolic responses to various substances were significantly upregulated (Figure 5E). These findings may indicate the importance of type I IFN in the regulation of hepatocyte immunity and intracellular metabolism in SARS-CoV-2 early infection.

## Discussion and Conclusion

The emerging pandemic of COVID-19 has severely impacted public health in the past year and a half, with tangible and terrible effects worldwide. In this context, this study focused on the transcriptional dynamics and infection characteristics of SARS-CoV-2 in cells from different backgrounds, thereby revealing some general patterns of SARS-CoV-2. Coronaviruses can evade IFN through various pathways, such as the nsp1 of SARS-CoV, which promotes host mRNA degradation and thus inhibits IFN production (Kamitani et al., 2006), or the ORF4a, 4b, and M proteins of the Middle East respiratory syndrome (MERS)-CoV, which inhibit IFN signaling by suppressing the IFN-stimulated response element (Yang et al., 2013). It has been clarified that SARS-CoV-2 has a stronger ability to inhibit IFN than SARS-CoV and the Middle East respiratory syndrome-CoV (Xia et al., 2020; Yao Z. et al., 2020), and many proteins (including ORF6, nsp1, nsp6, nsp13, nsp14, and nsp15) were reported to antagonize type I IFN (Sa Ribero et al., 2020; Thoms et al., 2020; Xia et al., 2020; Yuen et al., 2020). Herein, we focused on the pattern of combined intracellular expression of these IFN antagonist genes rather than on individual genes. This broader approach showed different types of up- or downregulation in Huh7 cells compared with Vero E6 and Huh7.5.1, which may be related to the ability to antagonize IFNs (Figures 1B, 5C). In addition to the viral genes mentioned earlier, several other endogenous type I IFN-sensitive viral genes were herein identified to potentially play a role in host–virus interactions, such as *nsp10* and *nsp16*, which can function as methyltransferases for 2′-O modification of viral mRNA to mimic the host capping process, implying that this capping modification may also help SARS-CoV-2 to evade IFN-mediated immune recognition or response (Daffis et al., 2010; Viswanathan et al., 2020). Furthermore, although there is still no experimental evidence to confirm the function or even the existence of *ORF10* (Michel et al., 2020), we sequenced the mature mRNA by capturing the polyA tail, and the collected data showed that *ORF10* and its upstream *N* gene were consistently expressed in high abundance during infection, presumably owing to their involvement in the regulation of viral genome replication. Noteworthy, the analysis approach applied in this study captured not only the subgenomic mRNAs of viral transcripts but also mixed viral genomic RNAs; thus, it was difficult to distinguish viral transcripts from viral replication using these data solely. We further examined the expression of some viral subgenomic RNAs in Vero E6 cells by qRT-PCR, revealing that both the inflection point of the expression trend (–ΔΔCt) (Supplementary Figure 10A) and the relative relationship between the *Ct* values of each subgenomic RNA (Supplementary Figure 10B) were consistent with the RNA-seq results (Figures 1B,D). Therefore, for the purpose of this study, the use of RNA-seq data to explain the transcriptional dynamics of SARS-CoV-2 is robust.

Focused on the host response to the different viral transcriptional stages, we were surprised to find that no significant immune activity was triggered in the host cells before the initiation of the second viral transcription (Figure 3, Supplementary Figures 6,7, and Supplementary Tables 6,7). The host immune system, especially in immortalized cell lines, is known to have a lagging response to pathogenic stimuli (Speranza and Connor, 2017); nonetheless, the herein observed lagged responses associated with viral transcription or infection stage were more suggestive of a hidden nature of SARS-CoV-2 when it invades the immune system of host cells. Therefore, we hypothesized that SARS-CoV-2 might suppress the innate immune system through its two-stage pattern in early infection; that is, the virus is latent in the first round of infection, and no immune response occurs in either IFN-deficient or normal host cells before it starts the second round. The host immune system is only significantly activated when the virus acquires a certain amount of accumulation and enters the second stage of logarithmic growth (Figure 2A). Although Huh7 cells were able to effectively restrict the viral processes, IFN-deficient Huh7.5.1 cells were not, and IFN-deficient Vero E6 cells even became culture vessels for SARS-CoV-2, indicating the need for type I IFN signals in early antiviral therapy. Notably, the metabolic activity of IFN-deficient Huh7.5.1 cells was significantly higher than that of Huh7 (Figure 5E). It has been shown earlier that IFN is able to resist viral infection by regulating intracellular metabolism (Wu et al., 2016). Thus, SARS-CoV-2 may use this approach to evade the IFN pathway.

Unlike other RNA viruses, the prominent host immune response is more likely to be resistant to bacterial infection than the typical IFN antiviral pathway, such as RIG-I, which is inhibited by the viral encoded protein (Rosenberg et al., 2018; O’Neal et al., 2019). This perspective opens two new questions: (i) Does the hidden nature of SARS-CoV-2 work by interfering with the pattern recognition receptor of the host? (ii) Does the host response more closely resemble bacterial-induced chronic infections due to inhibition of the IFN antiviral pathway? In particular, we noted that the IL-17 signaling pathway, which plays a key role in the pathogenesis of multiple chronic inflammatory diseases, was significantly upregulated in the second round of SARS-CoV-2 infection and may serve as a key mechanism to moderate inflammatory damage in COVID-19 (Durham et al., 2015; Maxwell et al., 2015). It should be noted that copper ions inhibited virus replication in both cells *in vitro* (Figure 2E), suggesting that the inhibition of SARS-CoV-2 by copper ions *in vitro* masked the cellular antiviral effects of metal ions in Huh7. Accumulation of copper ions may lead to increased oxidative stress and non-specific binding to macromolecules; therefore, most cells evolve complex copper regulation and transport systems that balance copper detoxification with copper acquisition (Ishida et al., 2013). However, the relationship between metal ion homeostasis and liver injury induced by SARS-CoV-2 infection needs to be further explored.

An effective way of combating self-limiting viruses is to identify host factors for targeted therapy (Ulrich and Pillat, 2020). Here, we propose three host genes that are highly associated with COVID-19: *CD38*, *PTX3*, and *TCN1*. It is unclear what role they play in COVID-19, but earlier studies have demonstrated that CD38 can act as a functional axis connecting IFN responses and respiratory syncytial virus-induced oxidative stress by inducing the production of local reactive oxygen species, thereby promoting the activation of inflammatory and antiviral processes (Lee et al., 2015; Wei et al., 2015; Schiavoni et al., 2018). In addition, CD38 is a marker of CD8^+^T-cell activation, with data showing high and sustained expression of CD38^+^HLA^–^DR^+^ and CD38^+^PD^–^1^+^ on CD8^+^ T-cells in fatal cases of COVID-19 (Xu et al., 2020; Zeng et al., 2020). These cases imply that inhibitors targeting CD38 may therapeutically alleviate the excessive inflammatory response caused by SARS-CoV-2. *PTX3* encodes an acute-phase protein involved in regulating inflammation and angiogenesis, which can bind influenza virus and cytomegalovirus, and subsequently inhibit viruses in multiple ways (Bottazzi et al., 2006; Reading et al., 2008). In the field of coronavirus, a protective effect of PTX3 against coronavirus-induced acute lung injury has been demonstrated (Han et al., 2012), and a recent study identified PTX3 as a new mortality biomarker for COVID-19 (Genç et al., 2020). Although these results imply the promise of PTX3 as a SARS-CoV-2 inhibitor, the dosing and clinical conditions need to be carefully evaluated to circumvent PTX3-induced adverse effects, such as unbalanced inflammatory response and endothelial dysfunction (Carrizzo et al., 2015; Magrini et al., 2016). Additionally, *TCN1* encodes a protein that is a major component of the secondary granules in neutrophils, suggesting that degranulation of neutrophils is strongly associated with SARS-CoV-2 infection (Stelzer et al., 2016; Akgun et al., 2020). Taken together, these three genes have distinct expression trends in clinical COVID-19 and non-COVID-19 cases and may serve as targets in immunotherapy or antiviral studies. It is necessary to further confirm biomarkers related to disease progression and determine the appropriate timing of interventional therapy.

Overall, mRNA as a product of the first step of biosynthesis reflects the earliest dynamics in host–virus interactions. Herein, dual transcriptional analysis of SARS-CoV-2 with different characterized hosts revealed the transcriptional dynamics of SARS-CoV-2 in the very early stages of infection, elucidating its “two-stages” infection profile and identifying the latency of intracellular immune response in the host, which may be related to disease progression and pathogenesis. Moreover, the IFN pathway was found to potentially contribute to the regulation of antiviral immunity and intracellular metabolism in hepatocytes. The limitation of this study is that the inferences obtained through data analysis have not been fully demonstrated by experimental means, but it is positive that these findings have been supported to varying degrees in other studies (Felgenhauer et al., 2020; Tian et al., 2020; Overmyer et al., 2021). In the future, more comprehensive and rigorous experimental designs should be carried out, combined with better analytical algorithms, to provide more reliable evidence for SARS-CoV-2 studies.

In conclusion, this study suggests potential mechanisms of viral infection and the function of some special host factors or type I IFN in host–virus interactions, providing new insights and targets for antiviral research and precision therapy.

## Data Availability Statement

The accession number for the raw sequencing data reported in this paper is CRA003624 (available at http://bigd.big.ac.cn). Count matrices and other original data are available from the corresponding authors on request.

## Author Contributions

WG and DL conceived and designed the experiments. YZ, ZC, and XH conducted the experiments. LJ, LM, and LW collected and analyzed the raw data. LJ, ZC, and YZ wrote the manuscript. HL and JC provided suggestions for writing. All authors discussed the results and commented on the manuscript.

## Conflict of Interest

The authors declare that the research was conducted in the absence of any commercial or financial relationships that could be construed as a potential conflict of interest.

## Publisher’s Note

All claims expressed in this article are solely those of the authors and do not necessarily represent those of their affiliated organizations, or those of the publisher, the editors and the reviewers. Any product that may be evaluated in this article, or claim that may be made by its manufacturer, is not guaranteed or endorsed by the publisher.

## Footnotes

1
https://www.pharmaceutical-technology.com/covid-19-vaccination-tracker/

## Supplementary Material

Supplementary Figure 1 Details of transcriptional patterns of SARS-CoV-2. **(a)** The expression curves of each viral gene at 0–24 hpi in Vero E6. **(b)** Expression curves of each viral gene at 0–24 hpi in Huh7. **(c)** Growth curves of each viral gene at 0–24 hpi in Vero E6. **(d)** Growth curves of each viral gene at 0–24 hpi in Huh7. **(e)** Immunofluorescence images corresponding to the time of appearance of the first peak and two troughs of the viral gene growth rate shown in Supplementary Figure 1. The bright-field was used as reference for cell density.

Supplementary Figure 2 DEGs shared by Vero E6 and Huh7 cells after infection by SARS-CoV-2. **(a)**, qRT-PCR validation of DEGs. Six DEGs with function in Vero E6 and Huh7 were selected for qRT-PCR detection and compared with the RNA-Seq results. Log2 (foldchange) of qRT-PCR is represented by –ΔΔCt. **(b)** DEGs shared by Vero E6 and Huh7 cells. Genes with opposite changes in the two cell lines are shown in red. **(c)** Expression of typical cytokines in the two cell lines.

Supplementary Figure 3 GO-terms semantic similarity of each group in Huh7 cells. Given the abundance and overlap of biological processes in each group of Huh7, we performed a GO-term semantic similarity analysis to calculate cell state correlations. *hclust* clustering revealed that the cellular states were more similar between 2 and 10 h and possessed distinct states between 12 and 24 h.

Supplementary Figure 4 Relation between gene co-expression modules and SARS-CoV-2 infection. **(a–e)** Weighted gene co-expression network analysis for Vero E6. **(f–i)** Weighted gene co-expression network analysis for Huh7. **(a,f)** Phylogenetic clustering tree of the genes. After identifying the gene modules according to the dynamic tree cut method, *hclust* was used to assign all the genes to their corresponding modules. **(b,g)** Pearson correlation between the module or genes with the trait (SARS-CoV-2 infection). **(c,h)** Functional enrichment analysis for each module. The Benjamini-Hochberg method was used to adjust the *P*-value, showing only the most central term. **(d)** Top 30 hub genes in the modules associated with infection. **(e,i)** Expression pattern of the hub genes in the modules correlated with infection. Log2 (foldchange) curves of genes at different time points are shown.

Supplementary Figure 5 Evaluation of the classification effect of the core genes on clinical data. **(a)** Principal coordinates analysis of 100 patients with COVID-19 and 26 patients with non-COVID-19. Clinical sequencing data from the Gene Expression Omnibus public database, accession number: GSE157103. **(b)** Receiver operating characteristic curves for the key genes.

Supplementary Figure 6 Independent weighted gene co-expression network analysis of Vero E6 corresponding to SARS-CoV-2 transcriptional rounds. The expression profiles of Vero E6 were split into two datasets for WGCNA analysis, corresponding to the first (0–14 hpi) and second (16–24 hpi) rounds of transcription of SARS-CoV-2, respectively. **(a,e)** Phylogenetic clustering tree of the genes. **(b,f)** Pearson correlation between the module with the trait (SARS-CoV-2 infection). **(c,d,g,h)** Function of the gene modules most associated with SARS-CoV-2 infection.

Supplementary Figure 7 Independent WGCNA analysis of Huh7 corresponding to SARS-CoV-2 transcriptional rounds. The expression profiles of Huh7 were split into two datasets for WGCNA analysis, corresponding to the first (0–10 hpi) and second (12–24 hpi) rounds of transcription of SARS-CoV-2, respectively. **(a,e)** Phylogenetic clustering tree of the genes. **(b,f)** Pearson correlation between the module with the trait (SARS-CoV-2 infection). **(c,d,g,h)** Function of the gene modules most associated with SARS-CoV-2 infection.

Supplementary Figure 8 Details of the transcriptional pattern of SARS-CoV-2 and enrichment analysis in Huh7.5.1. **(a,b)** Expression **(a)** and growth **(b)** curves of each viral gene at 0–24 hpi. **(c,d)** Enriched biological processes **(c)** and pathways **(d)** between 2 and 24 hpi.

Supplementary Figure 9 Correlations of *CD38*, *TCN1*, and *PTX3* with some immune markers in clinical patients. Clinical sequencing data from the GEO database, accession number: GSE157103.

Supplementary Figure 10 Comparison of methods for measuring the relative abundance of viral genes. **(a)** Determination of viral subgenomic RNA expression trends in Vero E6 by qRT-PCR. **(b)** Relative relationship between the *Ct* values of each subgenomic RNA largely consisted with the relative abundance of each open reading frame in RNA-seq.

Supplementary Table 1 Stat of sequencing data and gene counts matrices.

Supplementary Table 2 DEG lists and QPCR detection.

Supplementary Table 3 Top 10 GO-terms for each group.

Supplementary Table 4 Correspondence of co-expressed genes and modules.

Supplementary Table 5 TPM matrix from 126 clinical patients of 79 infection-related genes.

Supplementary Table 6 Phased WGCNA Results of Vero E6.

Supplementary Table 7 Phased WGCNA Results of Huh7.
