# Supplementary figures and images for: Suppression and Activation of Intracellular Immune Response in Initial Severe Acute Respiratory Syndrome Coronavirus 2 Infection
Source: Front Microbiol. 2021 Nov 26;12:768740. doi: 10.3389/fmicb.2021.768740 (PMC8661415; doi:10.3389/fmicb.2021.768740)

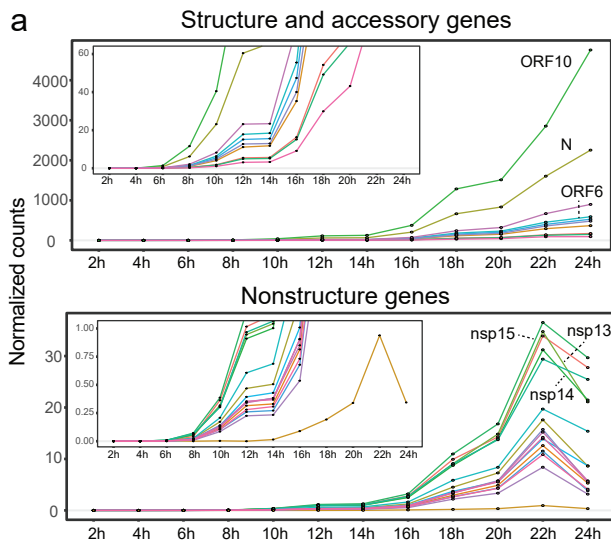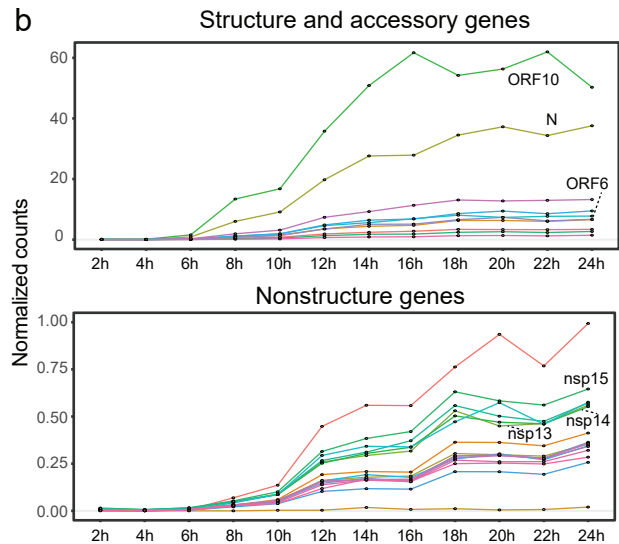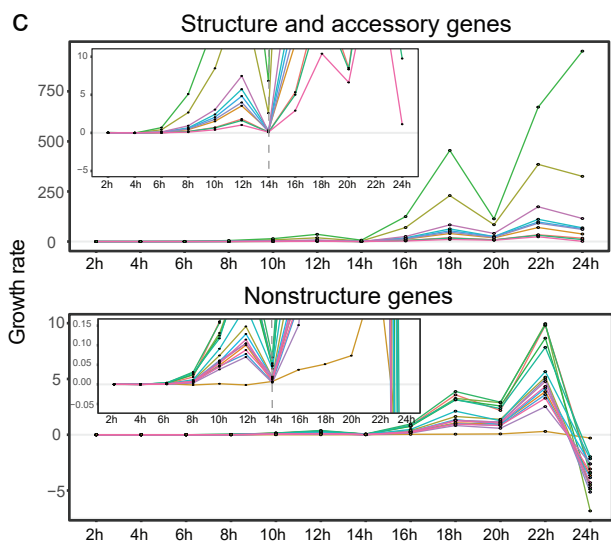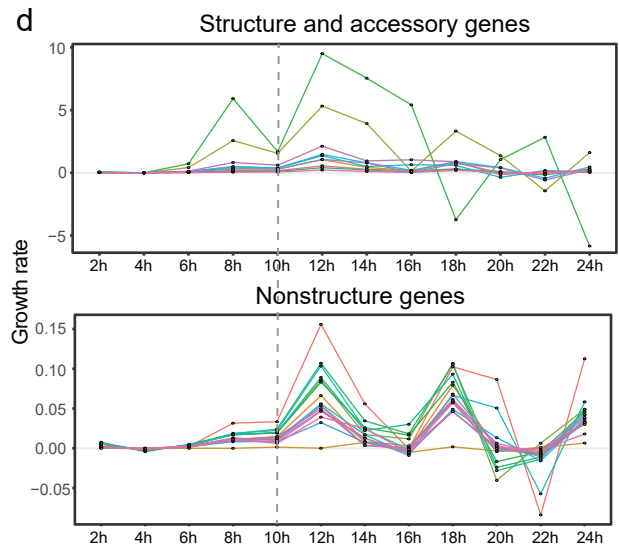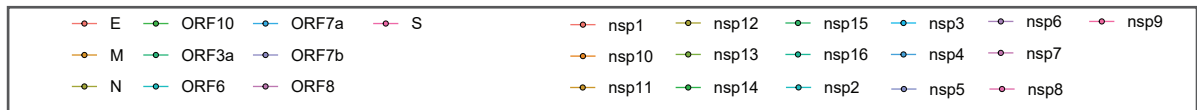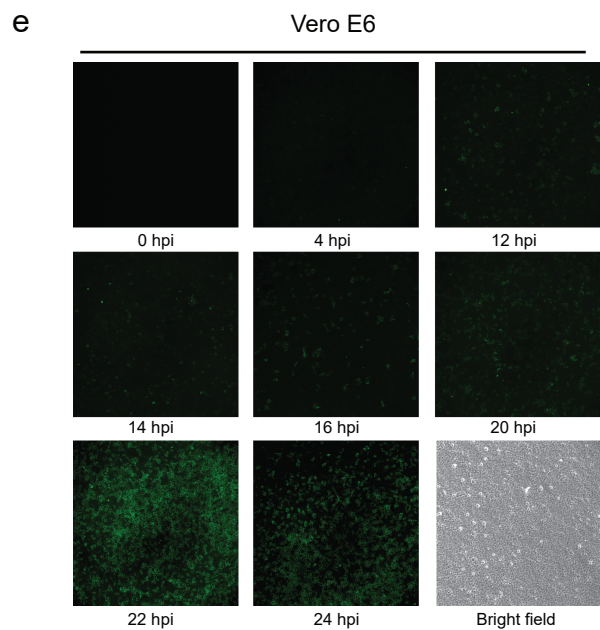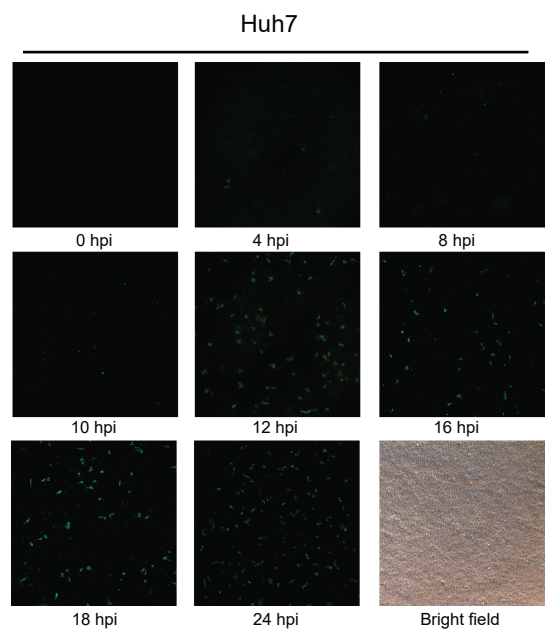

Supplement: Supplementary Figure 1 — Details of transcriptional patterns of SARS-CoV-2. (a) The expression curves of each viral gene at 0–24 hpi in Vero E6. (b) Expression curves of each viral gene at 0–24 hpi in Huh7. (c) Growth curves of each viral gene at 0–24 hpi in Vero E6. (d) Growth curves of each viral gene at 0–24 hpi in Huh7. (e) Immunofluorescence images corresponding to the time of appearance of the first peak and two troughs of the viral gene growth rate shown in Supplementary Figure 1. The bright-field was used as reference for cell density. [file Data_Sheet_1.PDF]

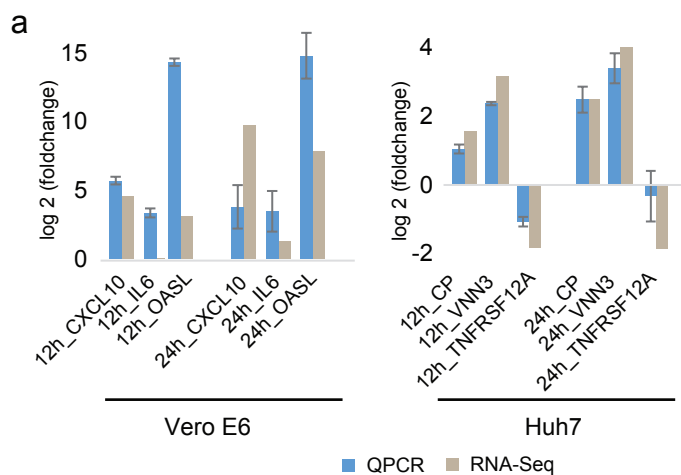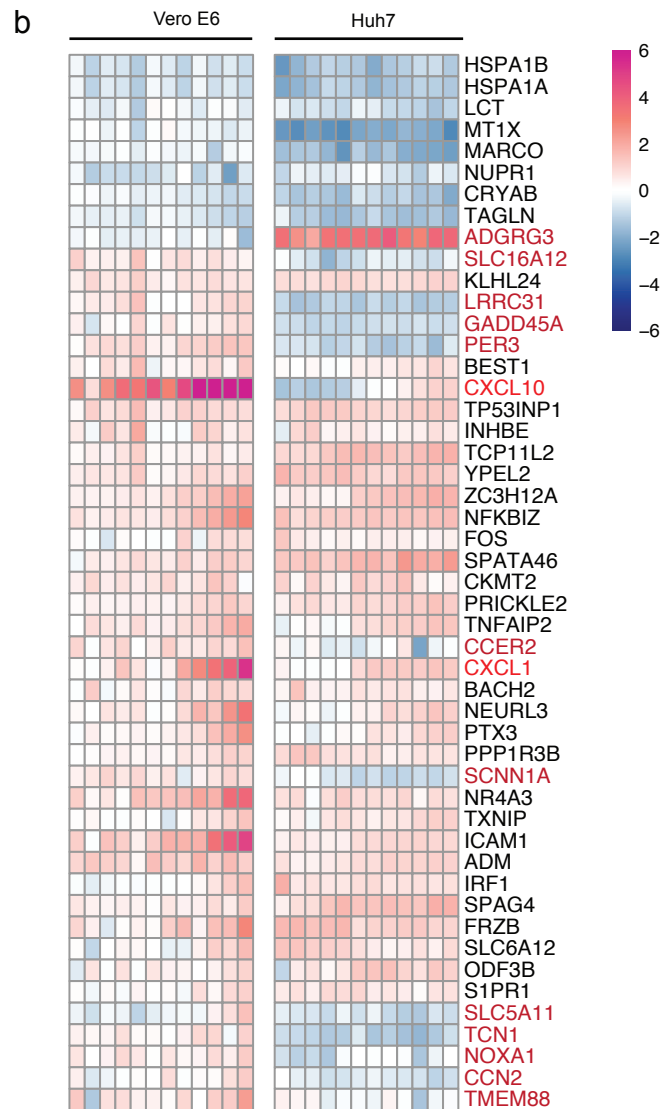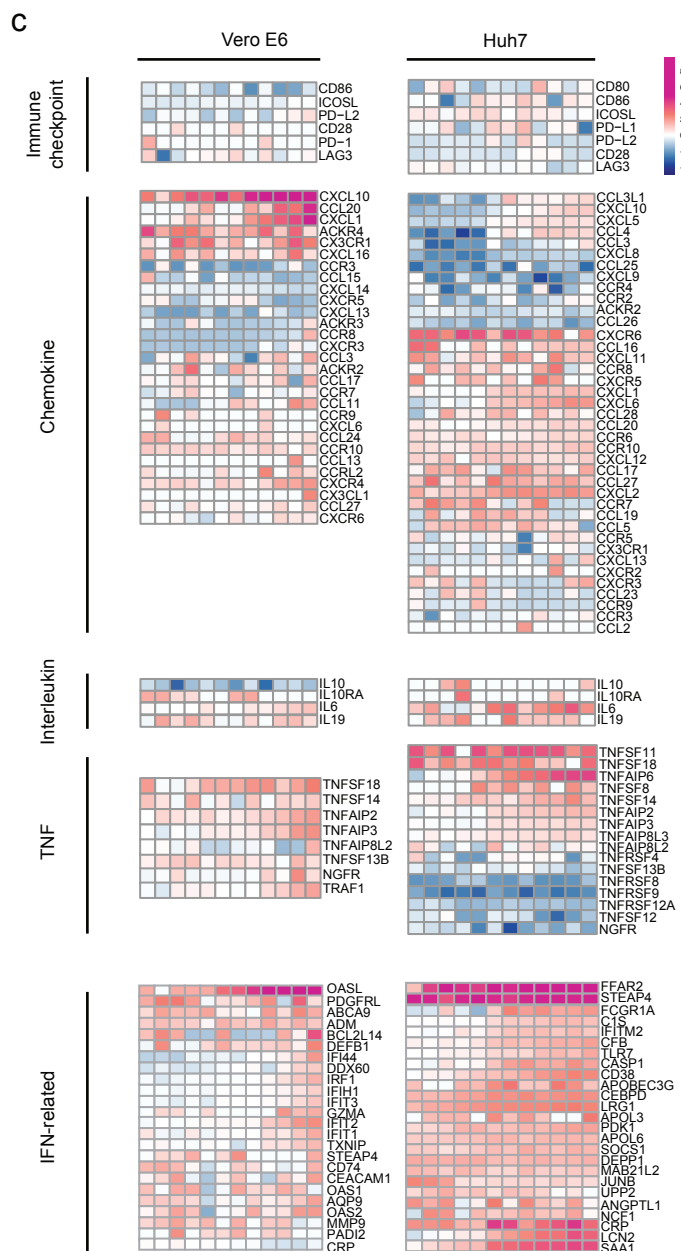

Supplement: Supplementary Figure 2 — DEGs shared by Vero E6 and Huh7 cells after infection by SARS-CoV-2. (a), qRT-PCR validation of DEGs. Six DEGs with function in Vero E6 and Huh7 were selected for qRT-PCR detection and compared with the RNA-Seq results. Log2 (foldchange) of qRT-PCR is represented by –ΔΔCt. (b) DEGs shared by Vero E6 and Huh7 cells. Genes with opposite changes in the two cell lines are shown in red. (c) Expression of typical cytokines in the two cell lines. [file Data_Sheet_2.PDF]

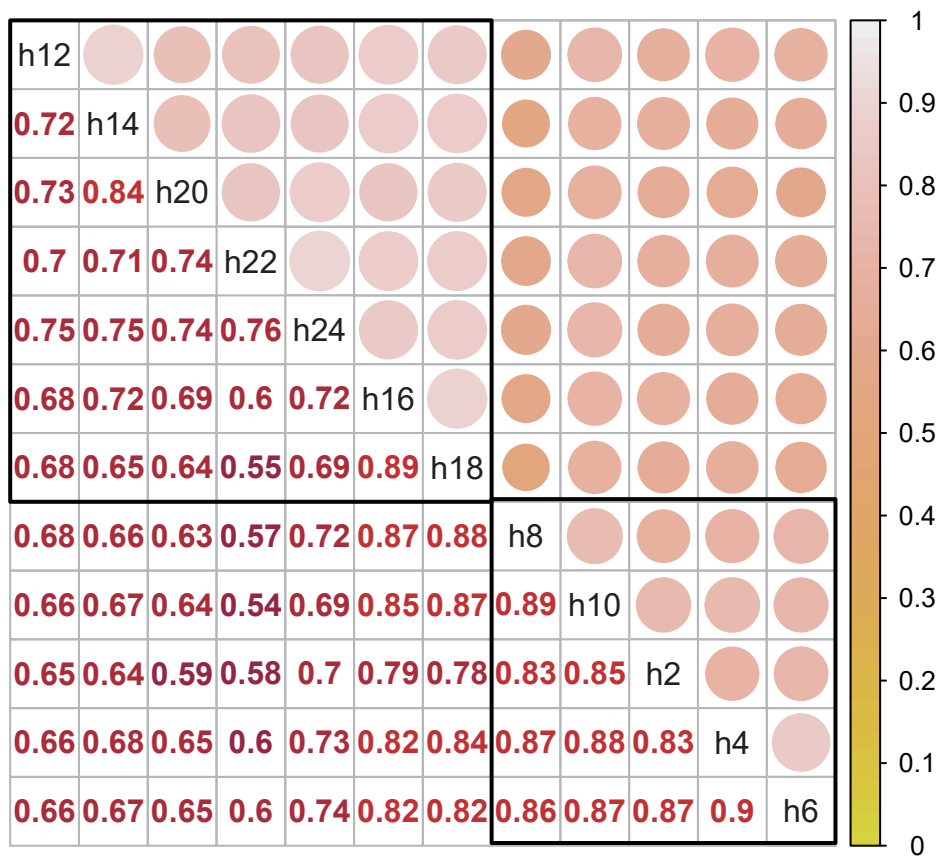

Supplement: Supplementary Figure 3 — GO-terms semantic similarity of each group in Huh7 cells. Given the abundance and overlap of biological processes in each group of Huh7, we performed a GO-term semantic similarity analysis to calculate cell state correlations. hclust clustering revealed that the cellular states were more similar between 2 and 10 h and possessed distinct states between 12 and 24 h. [file Data_Sheet_3.PDF]

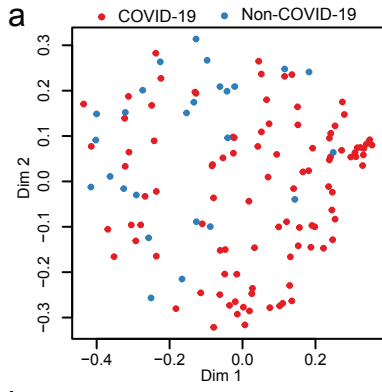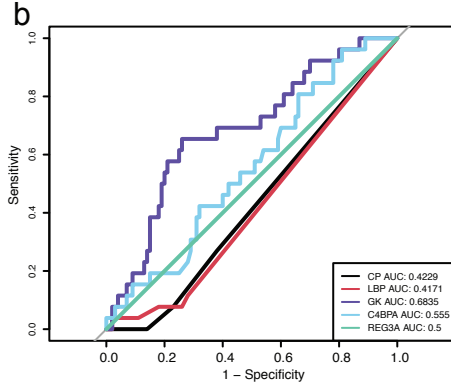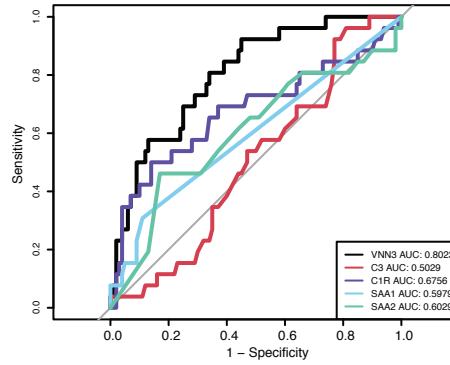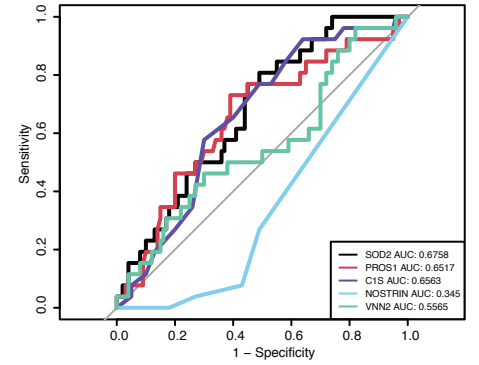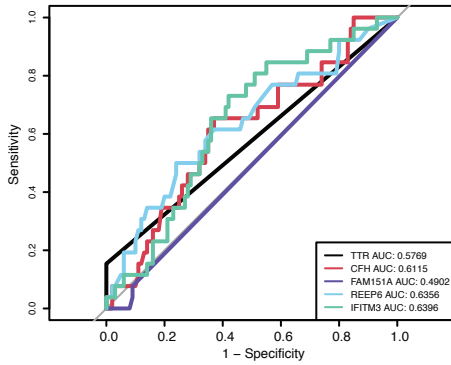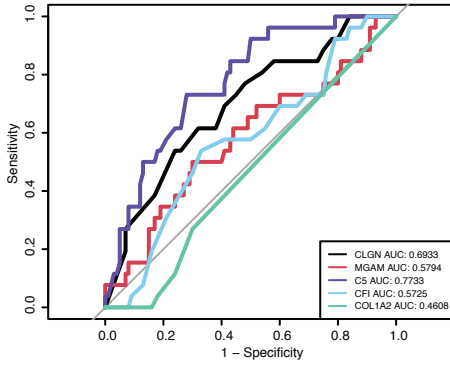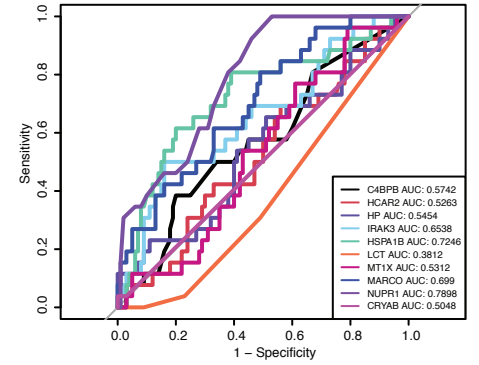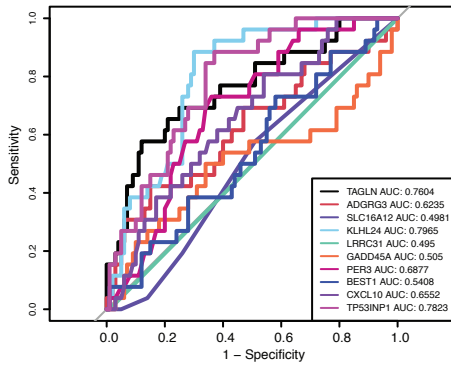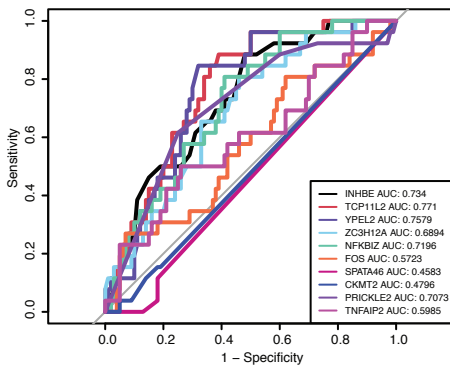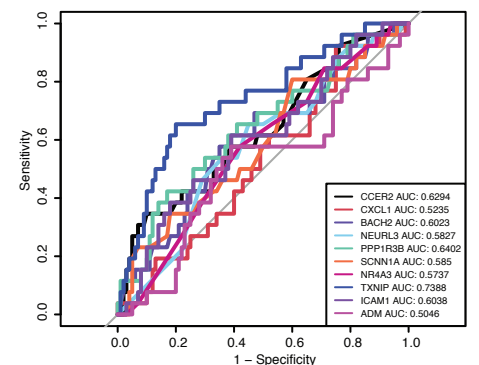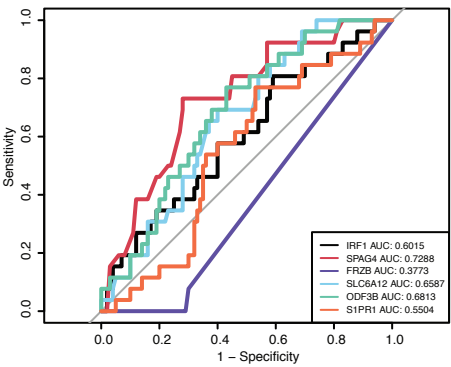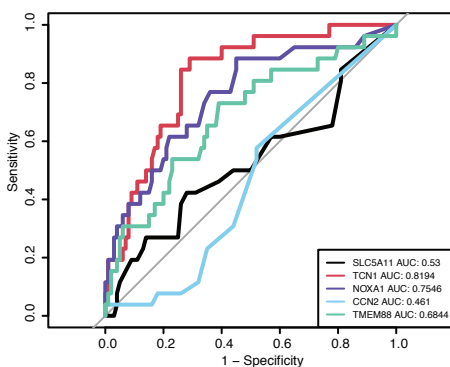

Supplement: Supplementary Figure 5 — Evaluation of the classification effect of the core genes on clinical data. (a) Principal coordinates analysis of 100 patients with COVID-19 and 26 patients with non-COVID-19. Clinical sequencing data from the Gene Expression Omnibus public database, accession number: GSE157103. (b) Receiver operating characteristic curves for the key genes. [file Data_Sheet_5.PDF]

Vero E6: stage 1 (0-14 hpi)

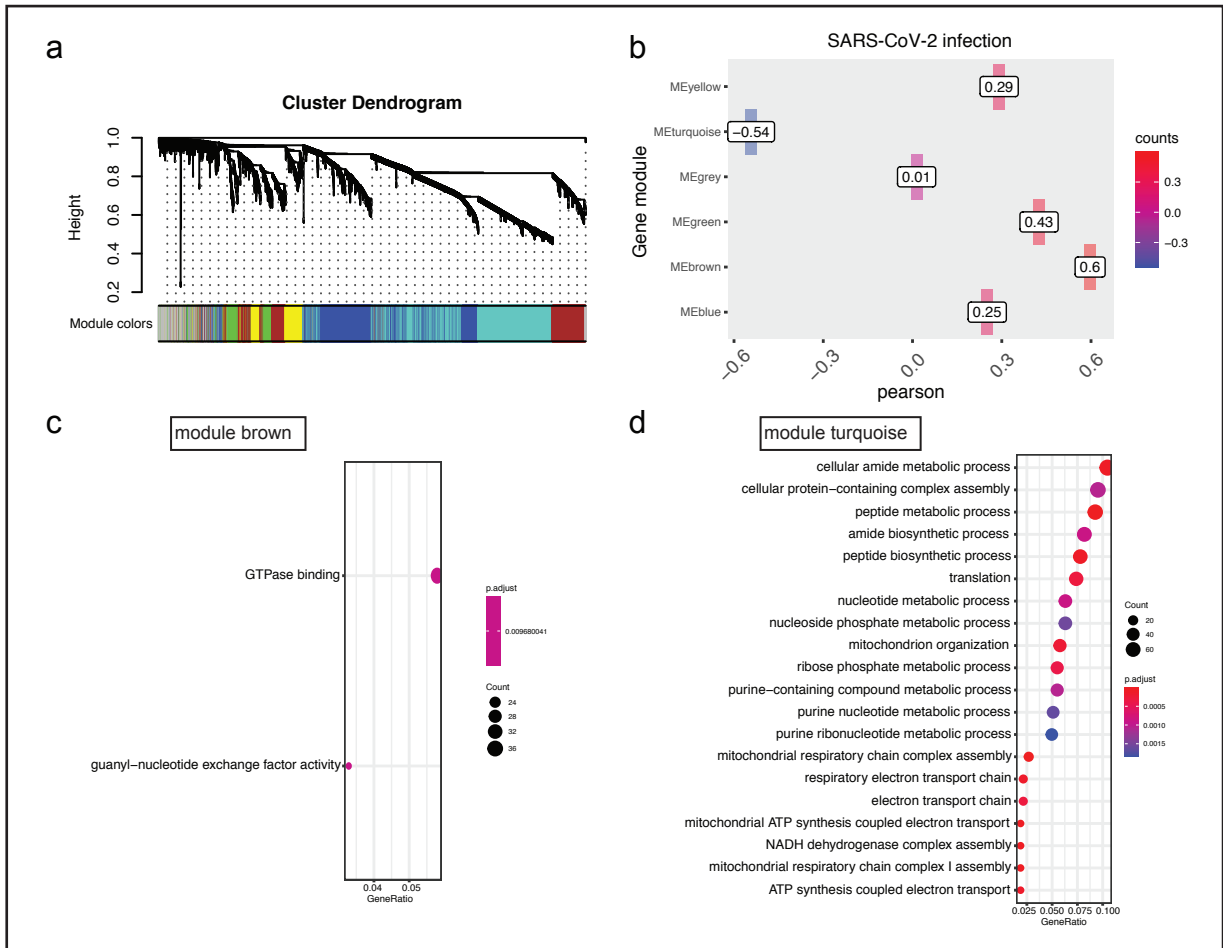

Vero E6: stage 2 (16-24 hpi)

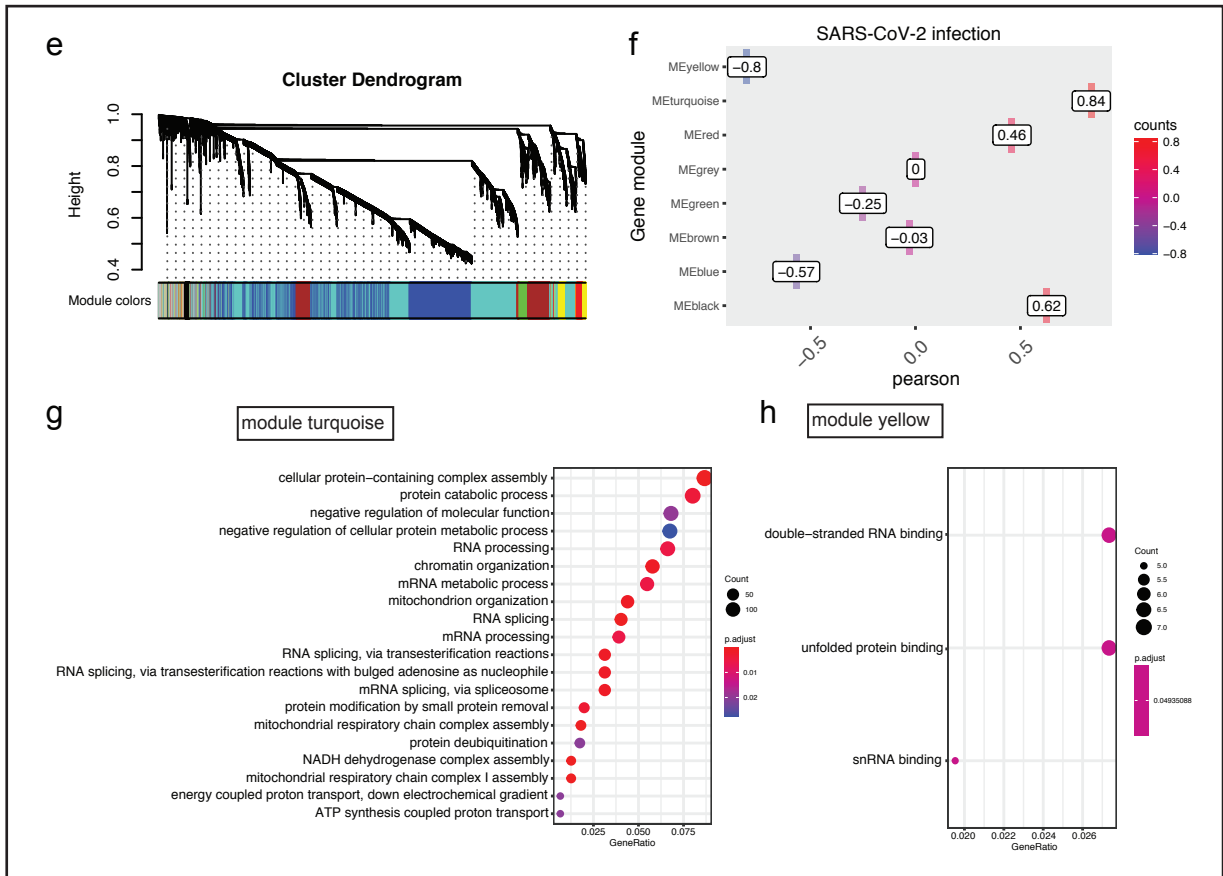

Supplement: Supplementary Figure 6 — Independent weighted gene co-expression network analysis of Vero E6 corresponding to SARS-CoV-2 transcriptional rounds. The expression profiles of Vero E6 were split into two datasets for WGCNA analysis, corresponding to the first (0–14 hpi) and second (16–24 hpi) rounds of transcription of SARS-CoV-2, respectively. (a,e) Phylogenetic clustering tree of the genes. (b,f) Pearson correlation between the module with the trait (SARS-CoV-2 infection). (c,d,g,h) Function of the gene modules most associated with SARS-CoV-2 infection. [file Data_Sheet_6.PDF]

Huh7: stage 1 (0-10 hpi)

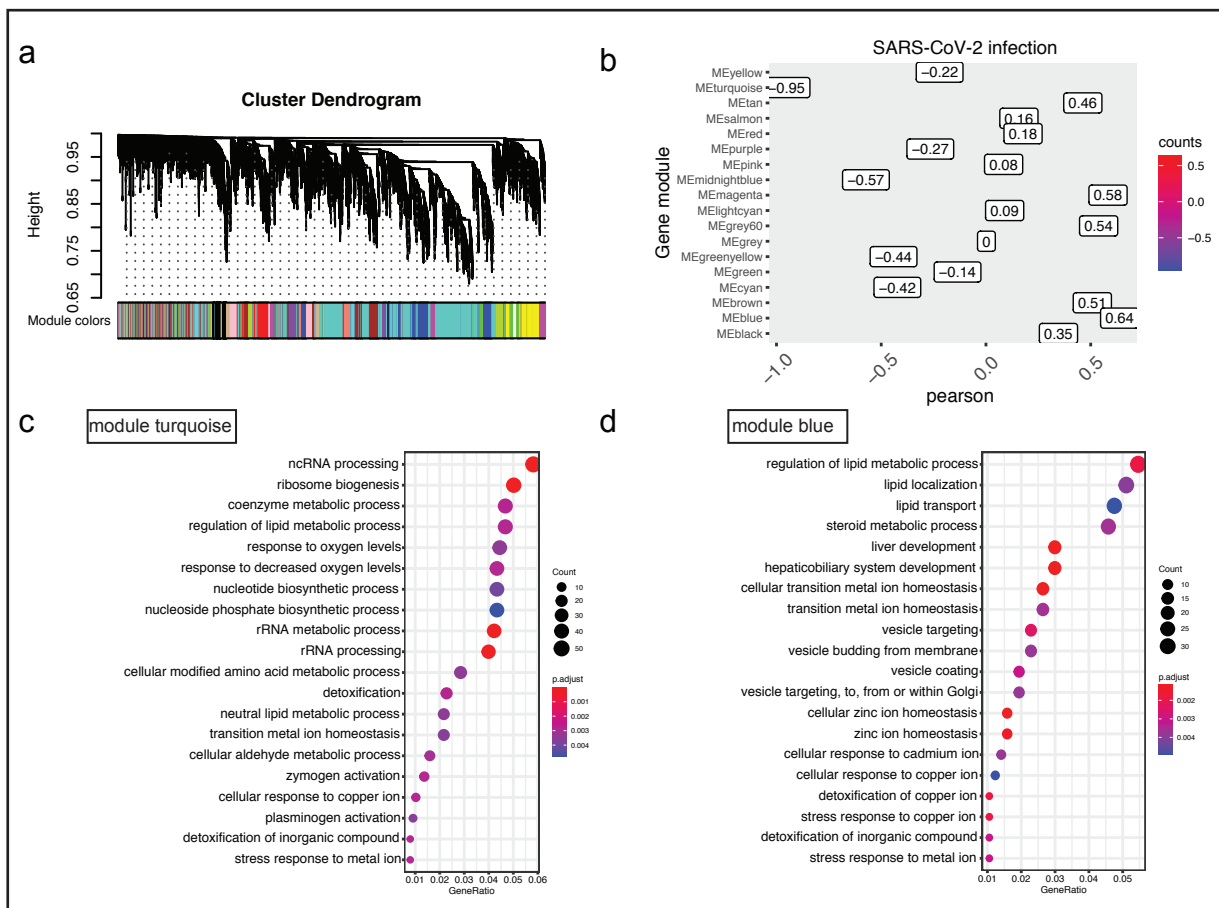

Huh7: stage 2 (12-24 hpi)

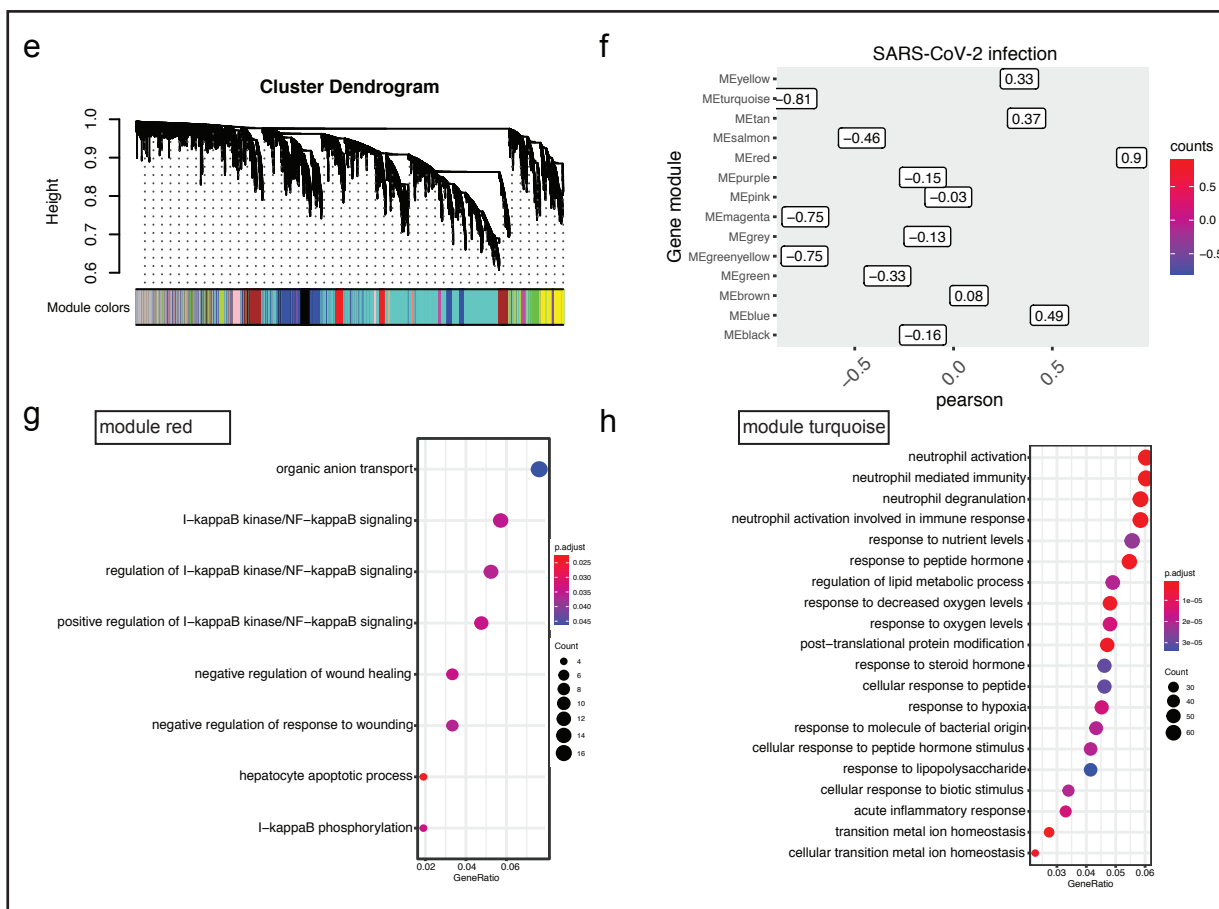

Supplement: Supplementary Figure 7 — Independent WGCNA analysis of Huh7 corresponding to SARS-CoV-2 transcriptional rounds. The expression profiles of Huh7 were split into two datasets for WGCNA analysis, corresponding to the first (0–10 hpi) and second (12–24 hpi) rounds of transcription of SARS-CoV-2, respectively. (a,e) Phylogenetic clustering tree of the genes. (b,f) Pearson correlation between the module with the trait (SARS-CoV-2 infection). (c,d,g,h) Function of the gene modules most associated with SARS-CoV-2 infection. [file Data_Sheet_7.PDF]

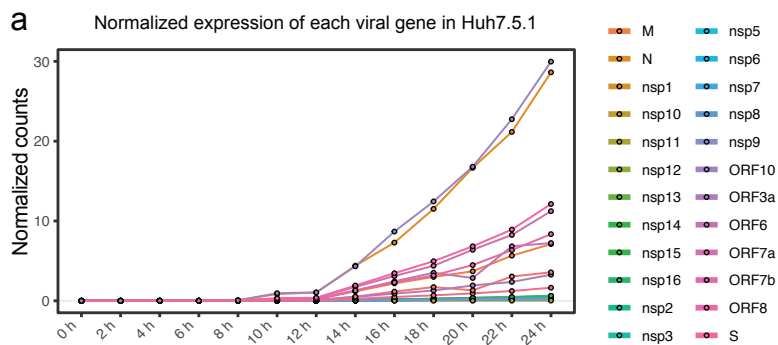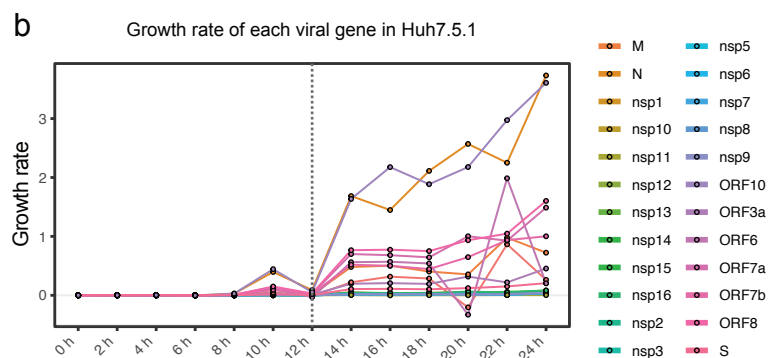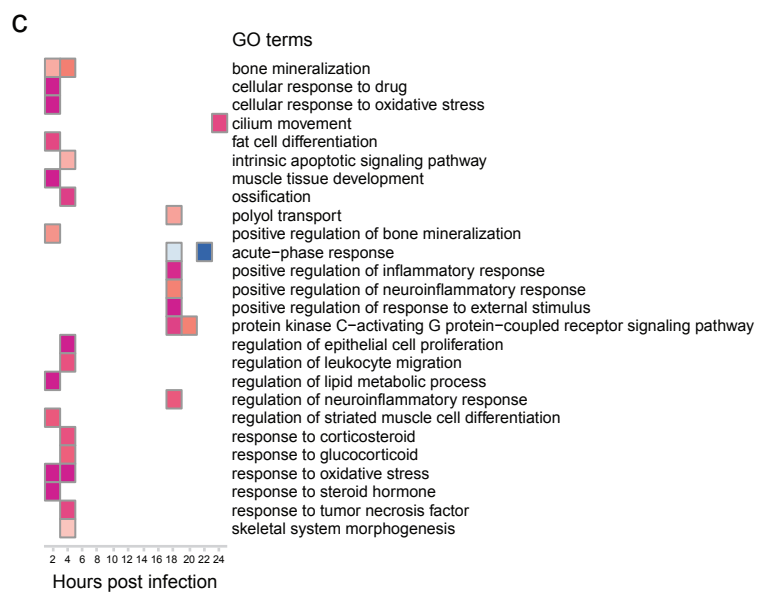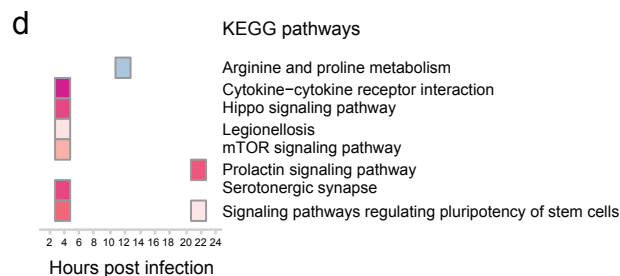

Supplement: Supplementary Figure 8 — Details of the transcriptional pattern of SARS-CoV-2 and enrichment analysis in Huh7.5.1. (a,b) Expression (a) and growth (b) curves of each viral gene at 0–24 hpi. (c,d) Enriched biological processes (c) and pathways (d) between 2 and 24 hpi. [file Data_Sheet_8.PDF]

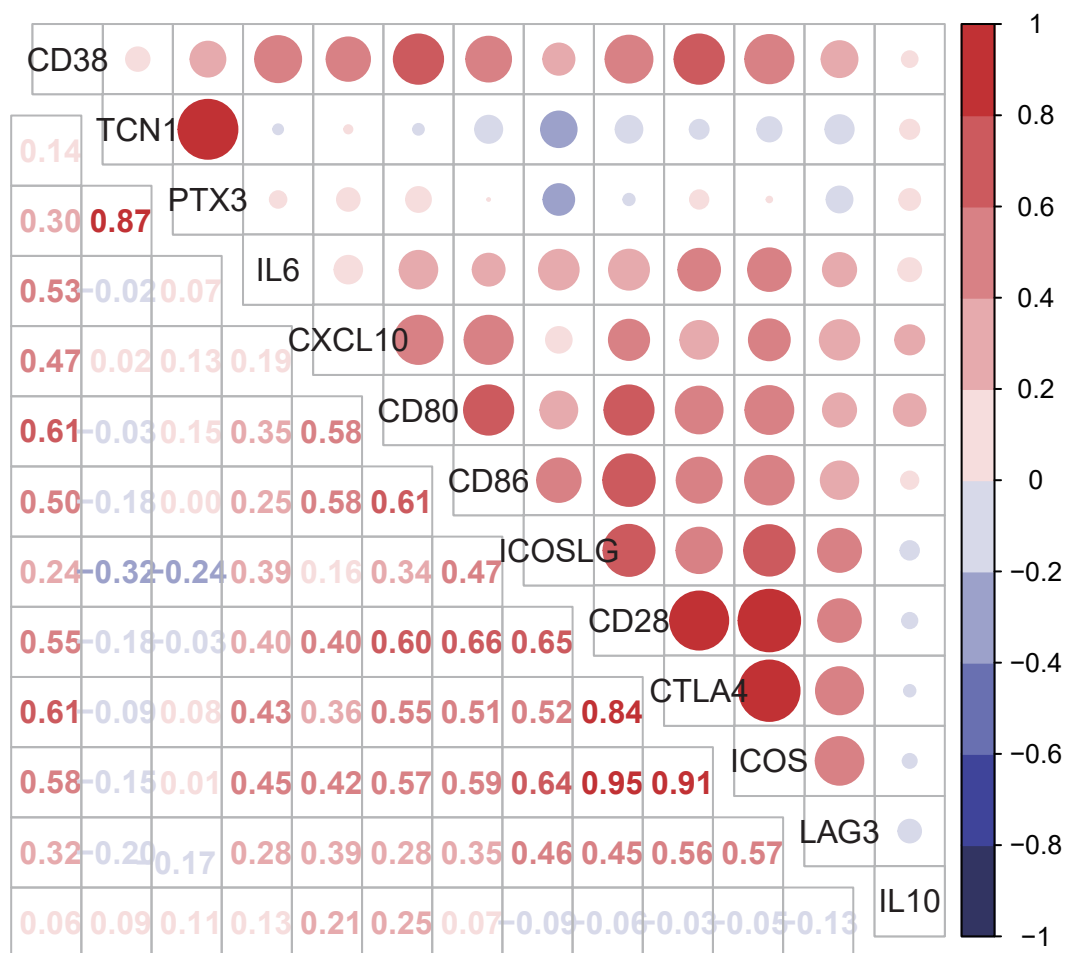

Supplement: Supplementary Figure 9 — Correlations of CD38, TCN1, and PTX3 with some immune markers in clinical patients. Clinical sequencing data from the GEO database, accession number: GSE157103. [file Data_Sheet_9.PDF]

a

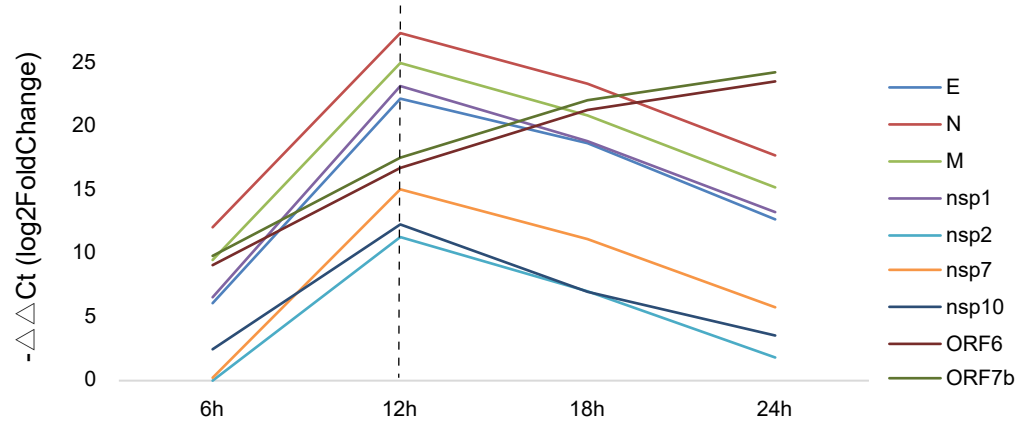

b

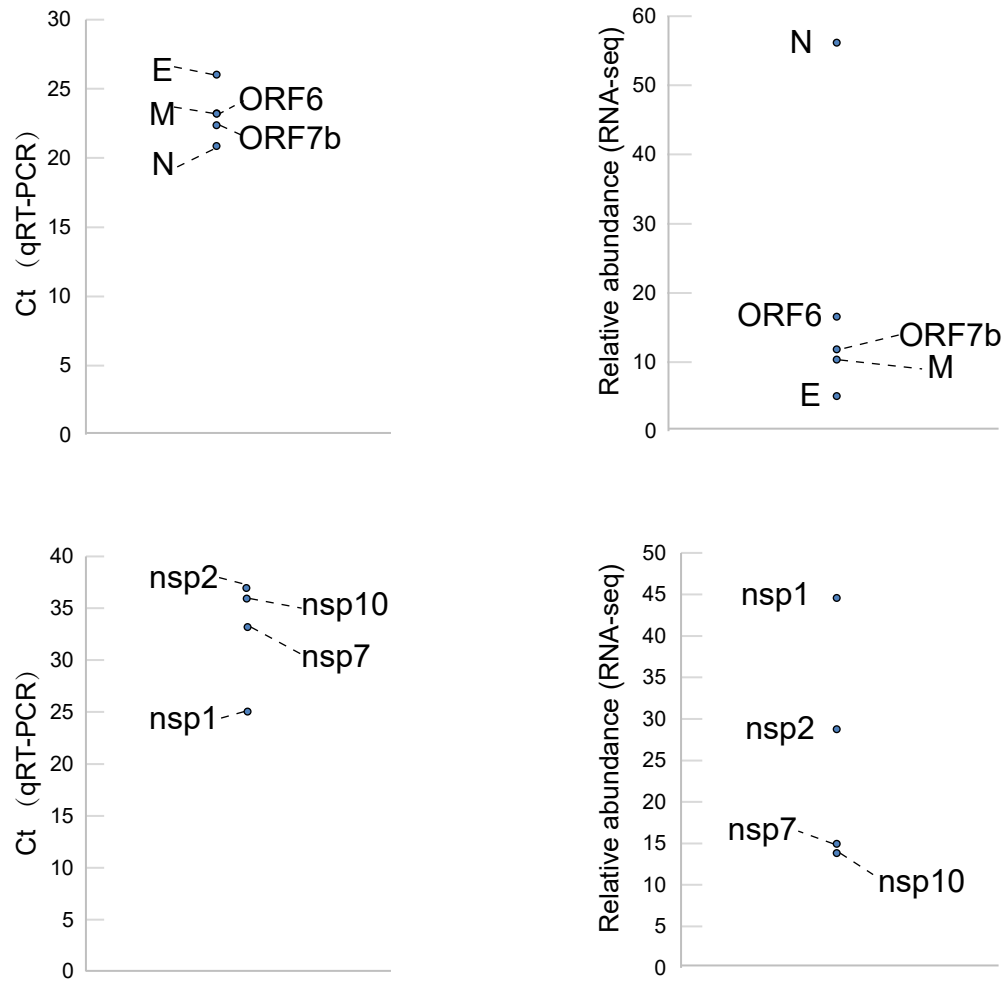

Supplement: Supplementary Figure 10 — Comparison of methods for measuring the relative abundance of viral genes. (a) Determination of viral subgenomic RNA expression trends in Vero E6 by qRT-PCR. (b) Relative relationship between the Ct values of each subgenomic RNA largely consisted with the relative abundance of each open reading frame in RNA-seq. [file Data_Sheet_10.PDF]
