# Supplementary material for: Suppression and Activation of Intracellular Immune Response in Initial Severe Acute Respiratory Syndrome Coronavirus 2 Infection
Source: Front Microbiol. 2021 Nov 26;12:768740. doi: 10.3389/fmicb.2021.768740 (PMC8661415; doi:10.3389/fmicb.2021.768740)

a

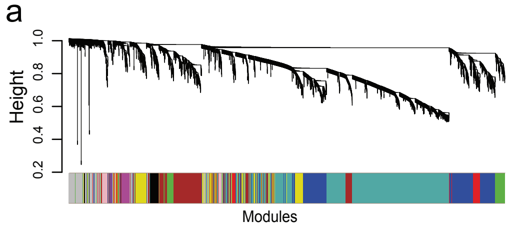

b

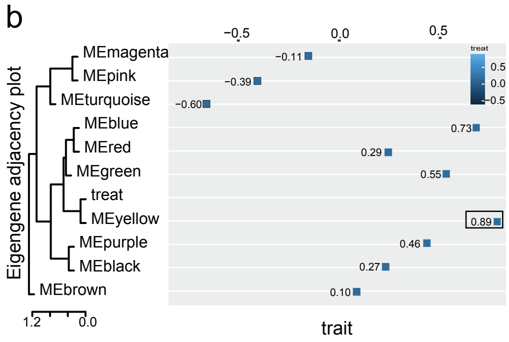

c

Module	Nr. of genes within the module	GO term	Benjamin-hochberg corrected p-value
black	185	none	
blue	1509	ubiquitin-protein transferase activity	11E-05
brown	1281	none	
green	406	none	
grey	712	gated channel activity	9.91E-07
magenta	153	none	
pink	174	DNA replication	1.75E-06
purple	54	none	
red	254	DNA metabolic process	1.63E-07
turquoise	2926	peptide metabolic process	1.92E-12
yellow	761	double-stranded DNA binding	3.43E-06

d

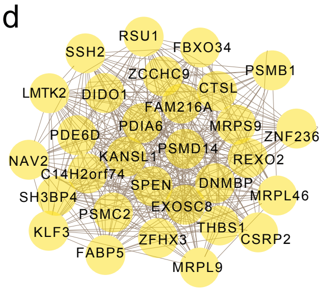

e

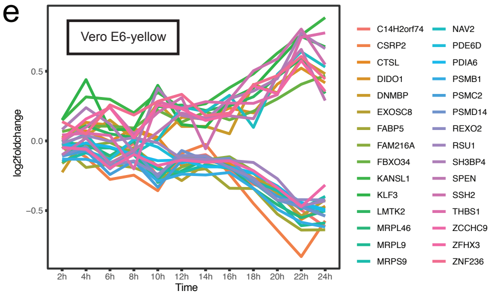

f

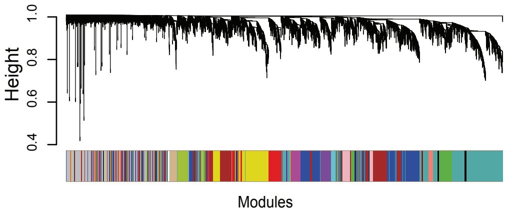

h

Module	Nr. of genes within the module	GO term	Benjamin-hochberg corrected p-value
black	395	none	
blue	2106	glycoprotein biosynthetic process	3.09E-06
brown	1722	covalent chromatin modification	1.01E-10
cyan	142	none	
green	672	NuRD complex	4.91E-06
greenyellow	310	positive regulation of tumor necrosis factor-mediated signaling pathway	1.83E-07
lightcyan	60	oxidative phosphorylation	2.37E-16
magenta	385	cholesterol biosynthetic process	1.35E-09
midnightblue	126	none	
pink	393	mitochondrial transmembrane transport	5.30E-07
purple	341	cardiac septum morphogenesis	4.57E-07
red	618	microtubule organizing center part	5.60E-08
salmon	159	cell leading edge	3.60E-05
tan	241	nuclear-transcribed mRNA catabolic process, nonsense-mediated decay	6.75E-17
turquoise	2562	acute inflammatory response	3.76E-11
yellow	1331	protein targeting to ER	1.97E-33
grey	1331	motile cilium	6.81E-08

g

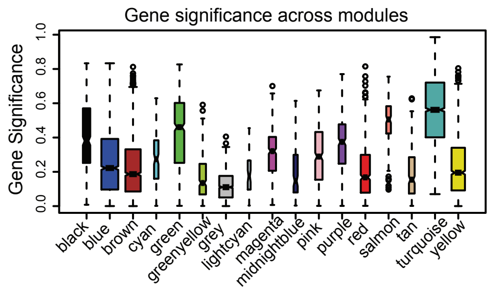

i

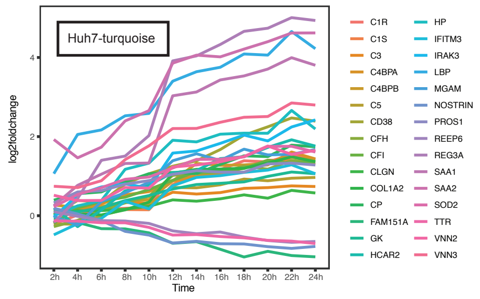

Supplement: Supplementary Figure 4 — Relation between gene co-expression modules and SARS-CoV-2 infection. (a–e) Weighted gene co-expression network analysis for Vero E6. (f–i) Weighted gene co-expression network analysis for Huh7. (a,f) Phylogenetic clustering tree of the genes. After identifying the gene modules according to the dynamic tree cut method, hclust was used to assign all the genes to their corresponding modules. (b,g) Pearson correlation between the module or genes with the trait (SARS-CoV-2 infection). (c,h) Functional enrichment analysis for each module. The Benjamini-Hochberg method was used to adjust the P-value, showing only the most central term. (d) Top 30 hub genes in the modules associated with infection. (e,i) Expression pattern of the hub genes in the modules correlated with infection. Log2 (foldchange) curves of genes at different time points are shown. [file Data_Sheet_4.PDF]
